# Copper-Loaded Layered Bismuth Subcarbonate—Efficient Multifunctional Heterogeneous Catalyst for Concerted C–S/C–N Heterocyclization

**DOI:** 10.1021/acsami.1c09234

**Published:** 2021-09-03

**Authors:** Marianna Kocsis, Sándor B. Ötvös, Gergely F. Samu, Zsolt Fogarassy, Béla Pécz, Ákos Kukovecz, Zoltán Kónya, Pál Sipos, István Pálinkó, Gábor Varga

**Affiliations:** † Department of Organic Chemistry, University of Szeged, Dóm tér 8, Szeged H-6720, Hungary; ‡ Materials and Solution Structure Research Group, and Interdisciplinary Excellence Centre, Institute of Chemistry, University of Szeged, Aradi Vértanúk tere 1, Szeged H-6720, Hungary; § Institute of Chemistry, University of Graz, NAWI Graz, Heinrichstrasse 28, Graz A-8010, Austria; ∥ Department of Physical Chemistry and Materials Science, Interdisciplinary Excellence Centre, University of Szeged, Szeged H-6720, Hungary; ⊥ Centre for Energy Research, Institute of Technical Physics and Materials Science, Konkoly, Thege M. út 29-33., Budapest 1121, Hungary; # Department of Applied and Environmental Chemistry, University of Szeged, Rerrich Béla tér 1, Szeged H-6720, Hungary; ¶ MTA-SZTE Reaction Kinetics and Surface Chemistry Research Group, Rerrich Béla tér 1, Szeged H-6720, Hungary; ∇ Department of Inorganic and Analytical Chemistry, University of Szeged, Dóm tér 7, Szeged H-6720, Hungary; ○ Department of Physical Chemistry and Materials Science, University of Szeged, Rerrich Béla tér 1, Szeged H-6720, Hungary

**Keywords:** bismutite, Sillen-type framework, Cu(II)-immobilization, cooperative bifunctional catalysts, heteroarylations, concerted C−N/C−S heterocyclization, synthesis of phenothiazines

## Abstract

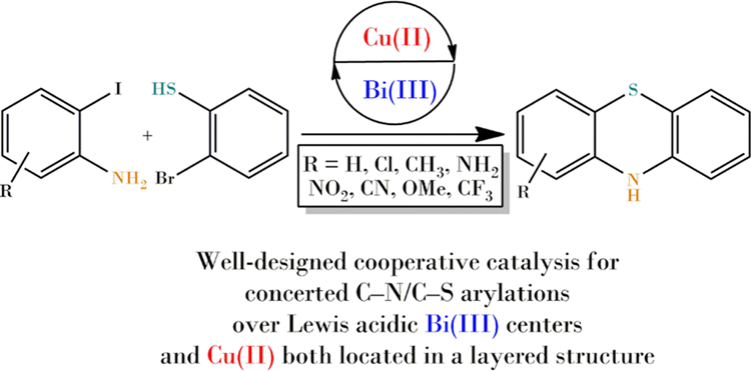

An efficient self-supported Cu(II)Bi(III) bimetallic catalyst with a layered structure was designed and developed. By careful characterization of the as-prepared material, the host structure was identified to exhibit a Sillen-type bismutite framework, with copper(II) ions being loaded as guests. The heterogeneous catalyst enabled C–N and C–S arylations under mild reaction conditions and with high chemoselectivities, thus furnishing valuable phenothiazines *via* heterocyclization with wide substrate tolerance. As corroborated by detailed catalytic studies, the cooperative, bifunctional catalyst, bearing Lewis acid sites along with copper(II) catalytic sites, facilitated an intriguing concerted C–N/C–S heterocyclization mechanism. The heterogeneous nature of the catalytic reactions was verified experimentally. Importantly, the catalyst was successfully recycled and reused multiple times, persevering its original structural order as well as its initial activity.

## 1 Introduction

Representative examples of N-heterocyclic structures with C–N and C–S moieties are phenothiazines and their derivatives. A diverse range of applications—from electrogenerated chemiluminescence emitters^1−3^ to chemosensors for selected fluorescence detection of special targets,^4−6^ or as molecular wires^7,8^—makes them highly valuable as intermediate materials or as active compounds. The phenothiazine backbone with various side chains can be used as an active ingredient of numerous psychotropic,^9^ antituberculous drugs,^10^ antifungal medication,^11^ inhibitors,^12,13^ or as a multiple drug resistance reverting agent.^14^ Although their applicability has been known for a long time, the actuality of phenothiazines is unquestionable, considering their novel applications are being continuously reported.^15−18^ For example, the piperazinyl phenothiazine antipsychotic agent, perphenazine was recently investigated against SARS-CoV-2 during clinical trials.^15^

Numerous different synthesis strategies of phenothiazines were drawn up in the past decades, including heat treatment of diphenylamines and sulfur at high temperature^19^ or a four-step route *via* Smiles rearrangement.^20^ Despite indisputable advances, the heterocyclization step continues to be insufficient. The most important challenge on this highlighted point is the realization of the ring-closing reaction by achieving a step-economical N–H/S–H functionalization cascade. Despite the fact that notable progress has been made by the design and development of powerful catalytic tools based on Ullmann-type couplings,^21−23^ many notable defects can be attributed to these strategies. Particularly, the application of a large amount of added base (10–30 mol %) as well the application of very long reaction time (48–96 h) and harsh conditions such as high reaction temperature (110–150 °C), sequential control of reaction conditions, and high catalyst loading (10–20 mol %) as well as possible side reactions coupled with moderate yields (50–70%) overcast the effectiveness of these systems. Of these, really few elegant, cascade processes were reported; however, using organic additives and/or high catalyst loading (30 mol %) is necessary to provide good yields (70–85%).^22,24^ Furthermore, while the focus has already been shifted toward the use of eco-friendly heterogeneous catalytic systems,^25^ no relevant progress has been achieved for producing phenothiazine in a heterogeneous catalytic manner. Additionally, contrary to noble metals and copper-based systems, capabilities of which are fully exploited, Lewis acids are mainly untapped. In view of the abovementioned weaknesses, it is surprising given that numerous modern C–S/C–N/C–C bond-forming reactions are based on Lewis acid-catalyzed coupling reactions as economical and ecologically benign alternatives.^26,27^ Conveniently, even though their application could be the means of solution for heterogeneously catalyzed concerted heterocyclization,^28,29^ based on Lewis acids and redox centers such as one of the most effective Cu(II) ions,^24,30−32^ at present, preparation of any cooperative catalyst is not known.

Previously, an alternative immobilization methodology for Cu(I) and Ag(I) ions was developed by us.^33−36^ With the aim of heterogenization of cations, a Sillen-type bismutite framework was applied as a host, furnishing a strong anchoring of the cations. As a result, novel layered type materials were prepared,^33^ which acted as robust, efficient, and selective catalysts for promoting Ullmann-type C–N coupling^34^ as well as for the direct synthesis of nitriles from terminal alkynes^35^ or the dehydrogenation of a wide scope of benzylic alcohols.^36^ Nevertheless, the active role of bismuth centers as Lewis acids, proving the bifunctionality of the as-prepared bismutite analogues, has not yet been harnessed.

Herein, the design and development of a bifunctional, bulk catalyst are reported on the basis of a Sillen-type bismuth-containing layered framework loaded with copper(II) species. The cooperative catalytic activity of the as-synthesized material was proven by means of cascade-like N-arylations and S-arylations of 2-iodoanilines and 2-bromobenzenethiol, yielding valuable phenothiazines *via* heterocyclization. Unlike in earlier cases,^23^ by taking advantage of bismuth and copper catalytic centers, simultaneous arylations were targeted, yielding concerted phenothiazine formation without the need for employing any extraneous ligand.

## 2 Experimental Section

### 2.1 Synthesis of the Copper-Containing Bismutite

In a typical synthesis, performed by the coprecipitation method, appropriate amounts of Bi(NO_3_)_3_·5H_2_O (1.82 g) and Cu(NO_3_)_2_·3H_2_O (0.91 g) were dissolved in 25 mL of 5 wt % nitric acid aqueous solution. After dissolution, 40–40 mL of 0.6–0.6 M ammonia and sodium carbonate solutions were added to the nitric acid solution containing the reagent salts and was stirred at 80 °C for 24 h. The obtained, colored product was filtered, washed with distilled water several times, and dried at 60 °C overnight. The desired product was marked as CuBi_2_O_2_CO_3_. For a comparison, copper-free bismutite (Bi_2_O_2_CO_3_) was also produced in the same way without loading the copper salt. For the same reasons, a bismutite-supported CuO composite (CuO@Bi_2_O_2_CO_3_) was also synthesized by a wet impregnation method previously reported.^37^

### 2.2 Characterization of the Copper-Containing Subcarbonate

The XRD patterns were recorded on a Rigaku XRD-MiniFlex II instrument by applying CuKα radiation (λ = 0.15418 nm) and 40 kV accelerating voltage at 30 mA.

The thermal behavior of the as-prepared layered composites was studied on a Setaram Labsys derivatograph. The instrument worked under constant air flow, and the heating rate was 1 °C/min. The samples, between 30 and 35 mg, were placed into high-purity alpha-alumina crucibles. To perform evolved gas analysis (EGA), a Pfeiffer QMS 200 mass spectrometer was used under oxygen flow (40 mL/min) with a 5 °C/min heating rate using ∼100 mg of the samples.

The structure-building inorganic components were identified by IR and Raman spectroscopy. Raman spectra were recorded with a Raman Senterra II (Bruker) microscope at an excitation wavelength of 765 nm by applying 12.5 mW laser power and averaging 20 spectra with an exposition time of 20 s. The instrument for recording the IR spectra was a Bio-Rad Digilab Division FTS-65A/896 (mid-range spectra) with 4 cm^–1^ resolution. The 4000–600 cm^–1^ wavenumber ranges were recorded. 256 scans were collected for each spectrum, in ATR mode by utilizing a Harrick’s single reflection diamond ATR accessory.

To determine the microstructure and oxidation state of copper, a combination of near-infrared (NIR), UV–vis, and XPS spectroscopies was used. NIR and UV–vis spectra were measured on a SHIMADZU UV-3600i Plus UV–vis–NIR spectrophotometer equipped with PMT, InGaAs, and PbS detectors in the 50,000–6000 cm^–1^ wavenumber range with 4 cm^–1^ resolution. Measurements were recorded in the reflection mode. The XPS measurements were carried out with a SPECS instrument equipped with a PHOIBOS 150 MCD 9 hemispherical analyzer, under a main-chamber pressure in the 10^–9^–10^–10^ mbar range. The analyzer was in fixed analyzer transmission mode with 40 eV pass energy for the survey scan and 20 eV pass energy for the high-resolution scans. The PB sample powder was pressed into an indium foil and loaded into the chamber on a gold-coated sample holder. The Al Kα X-ray source was used at 14 kV and at 150 W power. Charge referencing was done to the adventitious carbon (284.8 eV) on the surface of the sample. For spectrum evaluation, the CasaXPS commercial software package was used.

Detailed images from the morphology of the samples were gathered by a Philips CM20 instrument running at an acceleration voltage of 200 kV, and a Cs-corrected scanning/transmission electron microscope of Themis instrument was used. The TEM–EDS mapping was monitored by Super-X detectors of the Themis instrument at 200 kV. The SAED patterns were recorded and evaluated using ProcessDiffraction software.^38^ Porosity and surface area studies were performed on a NOVA3000 instrument (Quantachrome, USA) gas adsorption system using nitrogen as the adsorbate. Porosity data were calculated using the Barrett–Joyner–Halenda method in the 0.05–0.35 relative pressure range. All the samples were outgassed under vacuum for 16 h at 25 °C before adsorption measurements. The specific surface areas were measured by the BET method (adsorption of N_2_ at 77 K). The samples were flushed with N_2_ at 100 °C for 5 h to fully remove any adsorbents from the surface.

The amount of metal ions incorporated into the framework designed as well as potential leaching during the catalytic reactions were monitored by ICP-MS on an Agilent 7700× instrument. Before measurements, few milligrams of the samples measured by analytical accuracy were digested in 1.0 mL of concentrated nitric acid, and then, they were diluted with distilled water to 50 mL and then filtered.

### 2.3 Optimized Procedure for the Catalytic Reactions

The optimized procedure for the catalytic N- and S-arylation to produce phenothiazine is as follows. A mixture of DMSO and distilled water (1:2; 2 mL), the corresponding 2-iodoaniline or its derivatives (0.5 mmol, 1.0 equiv), 2-bromobenzenethiol (0.55 mmol, 1.1 equiv), K_2_CO_3_ as the base (2.5 equiv), and the copper-containing bismutite as the catalyst (19 mg, corresponding to 5 mol % metal ion loading) were combined in a nitrogen-flushed Schlenk-tube equipped with a magnetic stir bar. The reaction mixture was stirred at 90 °C for 15 h. Then, the mixture was cooled to room temperature, and the resultant liquid was extracted with brine (3 × 15 mL) and ethyl acetate (10 mL). The organic layer was dried over Na_2_SO_4_ and concentrated under reduced pressure. In order to find the mildest reaction conditions for the heterocyclization, the solvent, the temperature, the reaction time, the amount of the added base, and the catalyst loading were altered. Not only the activity but also the reusability of a potential catalyst was investigated in the heterocyclization reaction. The conversion and the selectivity were determined after each reaction by gas chromatography–MS (GC–MS) using a Thermo Scientific Trace 1310 Gas Chromatograph coupled with a Thermo Scientific ISQ QD Single Quadrupole Mass Spectrometer using a Thermo Scientific TG-SQC column (15 m × 0.25 mm ID × 0.25 μm film). During the measurements, parameters were as follows: column oven temperature: from 50 to 300 °C at 15 °C min^–1^; injection temperature: 240 °C; ion source temperature: 200 °C; electrospray ionization: 70 eV; carrier gas: He at 1.5 mL min^–1^; injection volume: 2 μL; split ratio: 1 to 33.3; and mass range: 25–500 *m*/*z*. Starting materials, products, and byproducts were identified using reference samples. The produced final products were also identified by NMR spectroscopy (listed in Section S4 in Supporting Information) upon using a Bruker DRX500 instrument 500 MHz NMR spectrometer. All samples were dissolved in 0.7 mL of DMSO-*d*_6_, and ^1^H NMR spectra were taken at room temperature. Spectra were fixed internally to the remaining resonance of the DMSO-*d*_6_ at 8.26 ppm.

## 3 Results and Discussion

### 3.1 Structural and Analytical Analysis of CuBi_2_O_2_CO_3_

**Figure 1:**
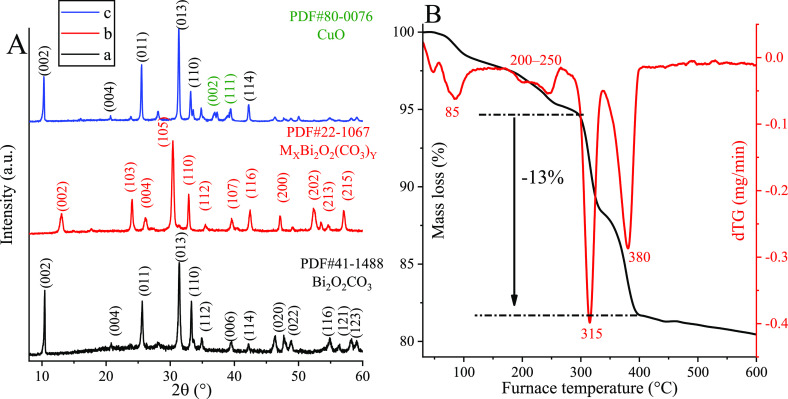
(A) XRD patterns of (a) Bi_2_O_2_CO_3_, (b) CuBi_2_O_2_CO_3_, and (c) 10% CuO on Bi_2_O_2_CO_3_ and (B) TG/DTG-curves of CuBi_2_O_2_CO_3_.

As shown in Figure 1, well-crystallized solids with primary particle sizes of about 17.0–43.0 nm, as calculated by the Scherrer equation, were produced by the abovementioned methods (Table S1). For both bismutite and CuO-modified bismutite, the diffraction peaks corresponding to the tetragonal structure with long-range order, analogous to that of bismuth subcarbonate (Bi_2_O_2_CO_3_) (PDF#41-1488),^39^ were detected at 10.5, 25.6, 31.4, and 33.2° 2θ positions with high intensities, indicating the formation of the bismutite structure with expanded interlayer gallery (0.665 nm → 0.791 nm) calculated by the Bragg equation (Table S1). On the basis of previous studies, it is known that carbonate ions and water molecules reside in the interlayer space resulting in an increase of the interlayer distance.^40^ Thermogravimetry/derivative thermogravimetry (TG/DTG) measurements on bismutite (Figure S1) verified this because 12.5% total weight loss was observed compared to a theoretical loss of 8.5%.^40^ In addition to the reflections originated from bismutite, the distinct peaks located around 37.0 and 39.5° corresponded to the (002) and (111) planes of monoclinic CuO (PDF#80-0076)^41^ grown over the surface-modified bismutite. By incorporating copper (ions) into the framework of Bi_2_O_2_CO_3_, diffraction lines appeared at 13.1, 24.1, 26.1, 30.4, and 32.9°. These reflections are related to the (002), (103), (004), (105), and (110) planes of an orthorhombic structure (PDF#22-1067)^42^ with smaller interlamellar space compared to that of bismutite. The cell parameters of the structure obtained resembles those of beyerite (Table S1).^43^ Beyerite belongs to the family of bismuth subcarbonates with a Sillen-like structure, in which intergrowth of [Bi_2_O_2_]^2+^ layers along with Ca^2+^ ions and (CO_3_)^2–^ layers are on top of each other. Consequently, copper species could be intercalated into the interlayer gallery of bismutite, causing the distortion of its tetragonal structure. As a result of the intercalation, a new orthorhombic phase could be solidified.

In order to learn about the accurate chemical composition of the beyerite-analogous structure, ICP-MS measurements (Table S1) combined with TG/DTG analysis (Figure 1B and Table S2) were performed. The actual Cu to Bi molar ratio of 0.25 was determined by ICP-MS. The TG curves exhibited four well-separated weight losses. The first two (50–85 and 200–250 °C) were attributed to the removal of water molecules. The first loss corresponds to weakly adsorbed water molecules on the outer surface of the material, while the second one belongs to the removal of interlayer water molecules, similarly to what was observed for layered double hydroxides.^44^ Incorporation of water molecules into the Sillen-type structure was not reported previously; their appearance is probably closely linked to the copper ions inserted into the framework structure. The other two weight losses can be assigned to the decomposition of carbonate ions and/or the hydroxyl groups intercalated among the layers. These observations are similar to those experienced with malachite, which is a hydroxyl-carbonate double salt of copper(II). Above 300 °C, both the hydroxyl groups and carbonate ions were lost exhibiting two mass losses in a relatively narrow temperature range (310–380 °C).^45^ This statement was verified by EGA with a TG–MS device. Complete dehydroxylation of our framework-modified bismutite took place by 350 °C, also verified by TG-MS; this observation is in good agreement with the weight loss previously reported for pure malachite.^45^ Upon increasing the temperature, the layered structure collapsed by 600 °C, and phase-pure, nonstoichiometric copper-bismuth mixed oxide was formed; this was confirmed by XRD (Figure S2; PDF#48-1886).^46^ The scale of the mass loss exhibited notable differences between the framework-modified bismutite and malachite because only one remarkable weight loss of pure bismutite (Figure S1)^40^ could be overlapped with double losses of malachite. Taking into account the measured actual molar ratio of metal ions as well as assuming a similar coordination sphere around copper ions to the microstructure of malachite, a possible composition for the product could be offered as Cu_0.5_Bi_2_O_2_(CO_3_)_1.25_(OH)_0.5_·*n*H_2_O. The theoretical weight loss of copper bismutite based upon this formula is 1.5% for the OH units strongly bound and 10% for CO_2_, providing a total of 11.5%. Measured weight loss of the composite in the range of 300–400 °C attributed to the decomposition of hydroxyl and carbonate groups is 13.0%, very close to the theoretical value.

**Figure 2:**
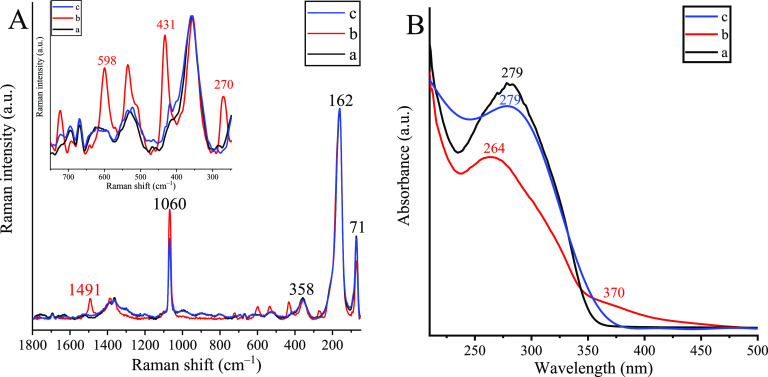
(A) Raman and (B) UV-DR spectra of (a) Bi_2_O_2_CO_3_, (b) CuBi_2_O_2_CO_3_, and (c) 10% CuO on Bi_2_O_2_CO_3_. The inset is a magnified section of (A).

Raman and UV-diffuse reflectance (DR) spectra of as-prepared composites evidenced both insertion of copper ions and their local structures convincingly (Figure 2). As can be observed in all Raman spectra, four relatively sharp, intense peaks represent the bismutite framework; two of them at 71 and 162 cm^–1^ can be assigned to the external vibrations of the [Bi_2_O_2_]^2+^ layer, one at 358 cm^–1^ is originated from Bi–anion stretching vibration mode, and the last one at 1060 cm^–1^ is related to ν_2_ out of plane bending mode vibration of the carbonate anions.^47,48^ By adsorbing 10% CuO on the surface of pure bismutite, no significant variations in the Raman spectrum were detected using a 785 nm excitation source. However, upon the incorporation of copper ions into the framework, the Raman spectrum displayed well-separated relatively intense peaks at 270, 431, 598, and 1491 cm^–1^, which can be regarded as a marker of copper ion insertion with malachite-like microstructure. Vibration frequencies below 600 cm^–1^ were directly assigned to stretching vibration modes of Cu–O (598 cm^–1^) and Cu–OH (431 cm^–1^) to the bending vibration mode of the OCu–OH unit (270 cm^–1^) in a malachite-like structure; these bands were found at positions almost identical to those of pure malachite.^49^ To make the assignation complete, the band at 1491 cm^–1^ was identified as the ν_3_ vibration band of the carbonate group, shifted from ∼1400 cm^–1^ detected in pure bismutite. These assignations mean further verification that the first coordination sphere of the incorporated copper is similar to that experienced in malachite. As was expected based on the Raman experiment, no remarkable changes in the optical properties of bismutite-supported CuO were detected by UV-diffuse reflectance spectroscopy (DRS) compared to pure bismutite. In contrast, the position of absorption maximum in the UV region shifted to lower frequencies for the framework-modified bismutite (279 nm → 264 nm). Moreover, a new absorption peak started to grow at 370 nm, which may be assigned to d–d electron transition between Cu(II) cations and CB electrons of bismuth subcarbonate.^50^

**Figure 3:**
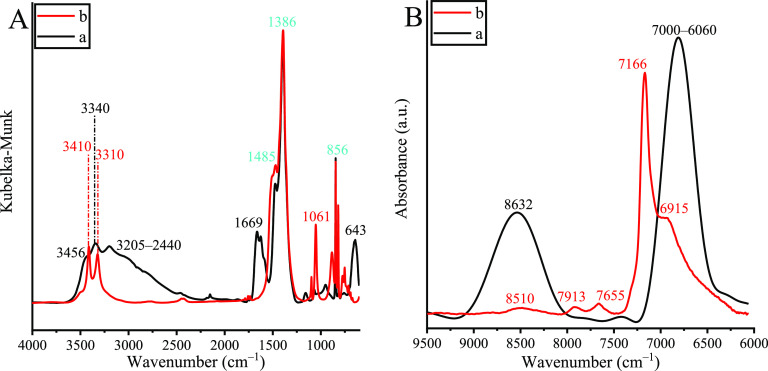
(A) Mid and (B) NIR spectra of (a) Bi_2_O_2_CO_3_ and (b) CuBi_2_O_2_CO_3_.

Mid-IR and NIR spectra of the pristine and the framework-modified bismutite (Figure 3) proved the different roles of the hydroxyl groups/water molecules in the structures. For the pristine sample, the water molecules are involved in a large-scale hydrogen-bonded system, while coordinating hydroxide anions are more probable for the framework-modified CuBi_2_O_2_CO_3_. Most of these intense absorption bands for each structure are found in the middle IR region (4000–600 cm^–1^), and they are mainly attributed to the stretching mode vibrations of the carbonate group [ν_1_: 836 cm^–1^; ν_3_(interlayer): 1386 cm^–1^ and *ν*_3_(surface): 1485 cm^–1^], which is consistent with that reported for bismutite previously.^48,51^ The band at 1061 cm^–1^ can be assigned to the *ν*_1_ stretching mode vibration of carbonate ions. The necessary condition for this vibration to be IR active is *D*_3h_ symmetry.^52^ This is possible if there is no interaction between (one portion of) the carbonate ions and the oxide layers. However, this band is lost after the successful intercalation of copper species, while a new absorption band at 643 cm^–1^ emerged ascribed to the symmetric stretching mode vibration of the Cu–O bond.^53^ This suggests that copper species replaced the weakly bound carbonate ions among the layers. Absorption bands that appeared in the region of 4000–2500 cm^–1^ are associated with the stretching vibrations of lattice water molecules and OH groups. In detail, the broad band around 3450 cm^–1^ belongs to the free or defect water that can readily overlap with the bridging and the H-bonded modes of water molecules located around 3300 and 3200–2500 cm^–1^. This assignment is capable of describing the complete OH region of the pristine bismutite reflecting the adsorption of water molecules.^48^ The formation of the M–OH bond is observed at 7166 and 6915 cm^–1^ for the framework-modified sample. These vibrations are accompanied by the appearance of very sharp, well-localized bands around 3300–3400 cm^–1^ without any significant broadening.^53,54^ On registering the region of 9500–7300 cm^–1^, further significant spectral differences attributed to electron transitions between the composites are seen. Nevertheless, the corresponding information is not yet available with the aid of which these differences can be interpreted.

**Figure 4:**
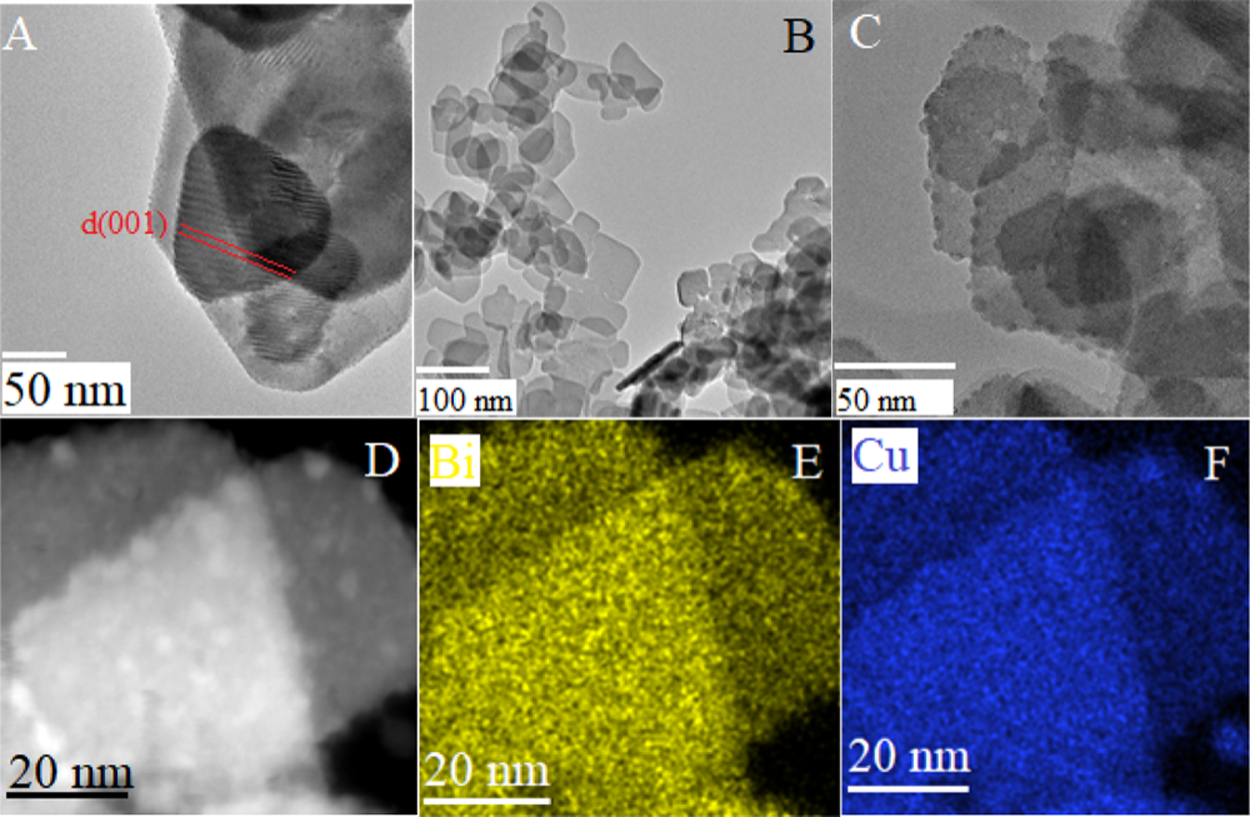
TEM images of (A) Bi_2_O_2_CO_3_ and (B,C) CuBi_2_O_2_CO_3_; TEM–EDX elemental maps of (D–F) CuBi_2_O_2_CO_3_.

Combined TEM/EDX (Figures 4 and S3) analysis also supported the formation of a typical Sillen-like layered structure with regular nanoplate-like morphology for the pure bismutite as well as the copper-loaded, surface-modified material. The samples were composed of monodisperse nanospheres with a uniform particle size of around 150 nm. The lattice spacings (*d*_001_) were estimated to be 0.661 and 0.785 nm for CuBiO_2_CO_3_ and Bi_2_O_2_CO_3_, respectively, which were in line with the calculated ones based on the X-ray diffractograms (Table S1). A very homogeneous copper (and bismuth) distribution was observed without measurable clusters or nonintegrated species.

**Figure 5:**
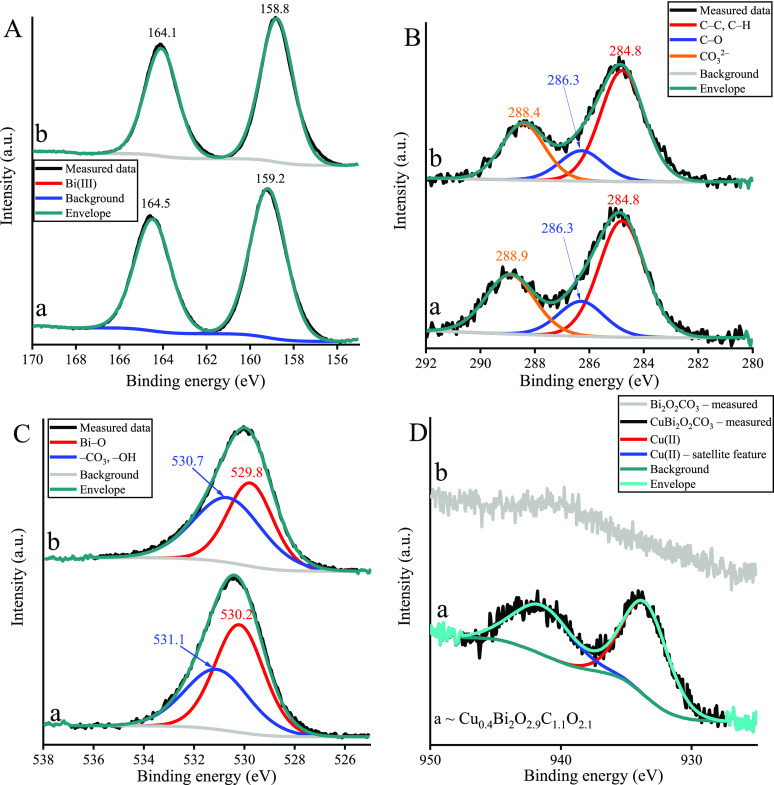
Bi 4f (A), C 1s (B), O 1s (C), and Cu 2p_3/2_ (D) XP spectra of CuBi_2_O_2_CO_3_ (a) and Bi_2_O_2_CO_3_ (b).

XPS studies were carried out to validate the information about the copper microstructure as well as to determine the surface compositions and chemical states on the bismutite structures. The survey scans showed the incorporation of the expected elements (Bi, C, O, and/or Cu) in the samples with a small amount of Na contamination for Bi_2_O_2_CO_3_ (Figure S4). Bi 4f spectra of the samples were fitted by only one component at this point (Figure 5). Both the measured binding energies and the separation of the 4f bands verified the presence of Bi(III) ions on the surfaces of the samples in both cases. It is worth mentioning that lower binding energy components were observable for the pure bismutite, as is expected on the basis of literature results.^40,55^ Additionally, it was also found that the significant shifts of the binding energies with the incorporation of the copper cations occurred, which reflected that the insertion of copper into the framework of bismutite occurred. The C 1s spectra of the samples were mainly composed of three regions. Generally, the binding energies at around 285 eV accounted for hydrocarbons, while the peak having binding energy above 286 eV was assigned to unidentified carbon–oxygen species. Moreover, there was further proof (∼288.5 eV) that carbonate ions from subcarbonate were also inserted into the structures. On copper insertion, notable shifts in the binding energy of carbonate–C 1s transition could be detected. It indicated a notable change of the interlayer gallery. Contrary to literature data, two components should only be used to fit the O 1s region of the samples. One of these has the characteristic binding energy for Bi–O bonding, while the second one could be attributed to Bi–CO_3_ linkage. The Cu 2p region of CuBiO_2_CO_3_ could be fitted with a 2p_3/2_ peak at 933.53 eV and a strong satellite feature at 941.42 eV. Accordingly, the exclusive presence of Cu(II) ions in identical chemical environments near the surface could be verified without any doubt. Overall, the surface analysis confirmed all of the abovementioned structural properties related to the bismutite-analogous structures, allowing us to unequivocally state the success of Cu(II) incorporation.^56^ Finally, the most probable surface composition of the framework-modified copper-bismutite composite—calculated from XPS data (Figure 5D)—was almost the same as was determined for the bulk with analytical measurements.

### 3.2 Application of CuBi_2_O_2_CO_3_ as a Heterogeneous Catalyst in Heterocyclization

**Scheme 1:**
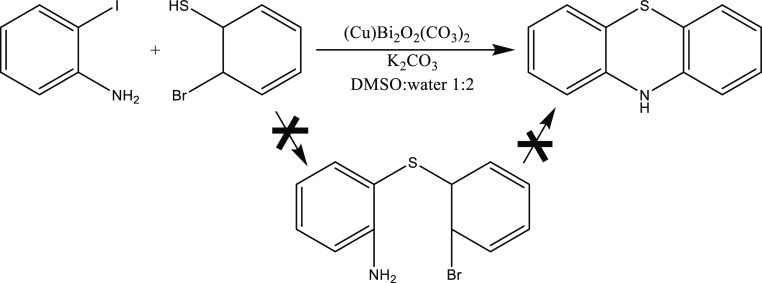
Possible Concerted Reaction for Phenothiazine Production with Simultaneous Formation of C–S and C–N Bonds From the Coupling Reaction of 2-Iodoaniline and 2-Bromobenzenethiol Over Bismutite and Its Cu(II)-Containing Derivatives

To probe the catalytic performance of the novel copper-containing bismuth oxide subcarbonate, heterocyclization model reaction was chosen. During the process, new C–S and C–N bonds were constructed over bismutite and the composites were applied (Scheme 1). The yields and the selectivities were used as indicators.

**Figure 6:**
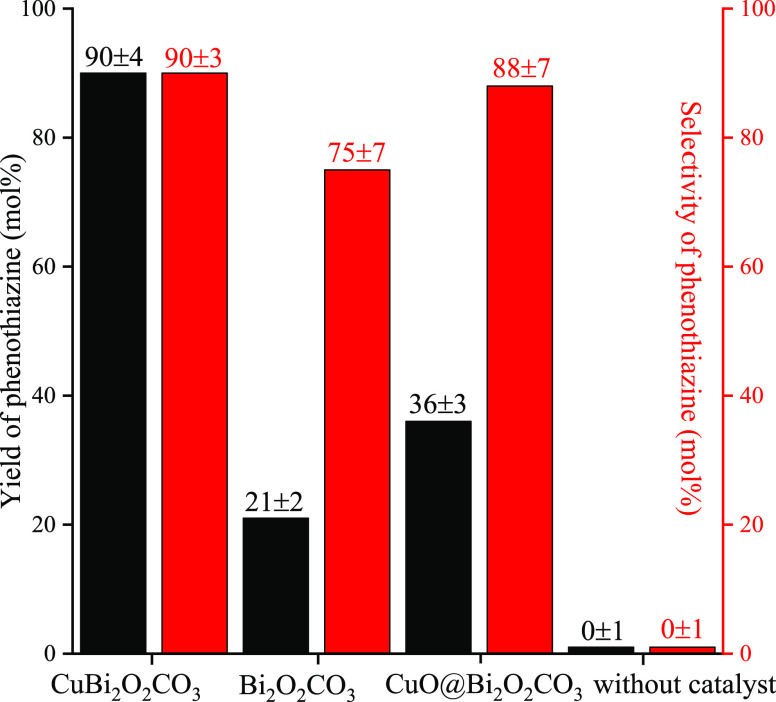
Effect of the quality of the catalysts in the cascade-like C–S and C–N heterocyclization reaction to produce phenothiazine (see Scheme 1). Reaction conditions: 1 equiv (0.25 M) of 2-iodoaniline, 1.1 equiv of 2-bromobenzenethiol, 5 equiv of K_2_CO_3_, 10 mol % catalyst, 90 °C for 24 h, 110 °C for further 48 h.

Our scouting experiments were performed under conditions similar to those used by Ma and co-workers in an earlier study.^23^ While the conversion of 2-iodoaniline was zero under catalyst-free conditions, a competitive reaction could be seen producing sulfur-containing byproducts *via* a thermal pathway (Figure 6). The framework-modified CuBi_2_O_2_CO_3_ proved to be an efficient catalyst, producing phenothiazine with high yield (90%) and high selectivity (90%). The catalyst provided the desired product in DMSO without employing any cocatalytic additives or ligands, the unavoidable presence of which was previously demonstrated in homogeneous catalytic processes.^22,24^ In the thermal pathway, 10 mol % sulfur-containing byproducts, mostly dibenzotiophene, were found to be formed in the homocoupling side reaction of 2-bromobenzenethiol. Similarly, decreased yield characterized the CuO@Bi_2_O_2_CO_3_ system as well. On using Bi_2_O_2_CO_3_ as the catalyst, Bi(III) centers exhibited catalytic activity toward the formation of phenothiazine but only with a moderate yield of 21% and reduced selectivity of 75%. It is not unprecedented that Bi(III) having a well-known Lewis acid character can act as a catalyst in heterocyclization reactions.^57,58^ Nevertheless, to the best of our knowledge, there is no relevant information about using heterogeneous bismuth catalysts for promoting similar reactions.

**Figure 7:**
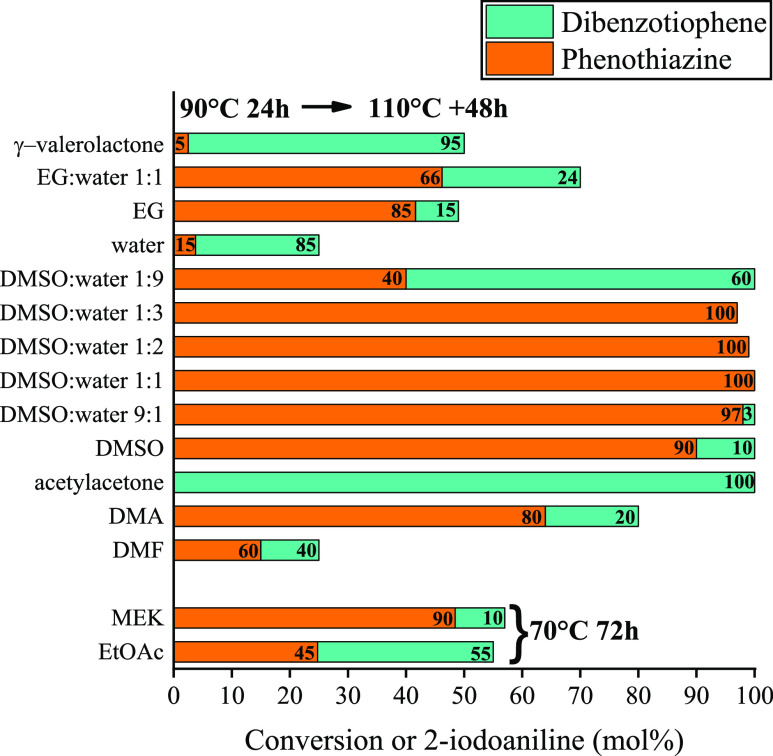
Effect of various solvents in the concerted C–S and C–N heterocyclization in producing phenothiazine (see Scheme 1). Reaction conditions: 1 equiv (0.25 M) of 2-iodoaniline, 1.1 equiv of 2-bromobenzenethiol, 5 equiv of K_2_CO_3_, 10 mol % CuBi_2_O_2_CO_3_, 90 °C for 24 h, 110 °C for further 48 h or 70 °C for 72 h.

**Figure 8:**
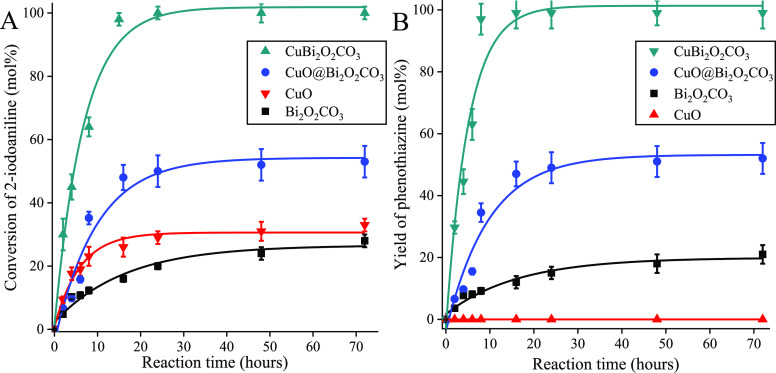
Conversions of 2-iodoaniline (A) and yields of phenothiazine (B) as the function of time in the concerted C–S and C–N heterocyclization producing phenothiazine (see Scheme 1) catalyzed by various Bi- and/or Cu-containing catalysts. Reaction conditions: 1 equiv (0.25 M) of 2-iodoaniline, 1.1 equiv of 2-bromobenzenethiol, solvent: DMSO/water 1:2, 2.5 equiv of K_2_CO_3_, 2.5 mol % catalyst, 90 °C.

The application of solvents^59^ other than DMSO led to a notable decrease in the phenothiazine yield except for solvent mixtures of DMSO/water (Figure 7). A 1:1 mixture of these behaved extremely well, both the conversion and the selectivity reached 100%. This finding can be considered as particularly promising, if one takes into account that nonpolar and aprotic solvents with major issues (THF, diethyl ether) proved to be appropriate generally. The unique activity of CuBi_2_O_2_CO_3_ made it possible to drop the reaction temperature and decrease the reaction time. The heterocyclization was found to proceed to completion with exclusive selectivity toward the desired product at 90 °C in 72 h using 5 mol % CuBi_2_O_2_CO_3_ as a catalyst and a 2.5 equiv base (Figures S5 and S6). Notably, there is no relevant information on using a similarly low concentration of base to yield phenothiazine exclusively as optimal, as found in the present case. Furthermore, it became clear that the outstanding activity of the catalyst allowed for the reduction of the reaction time, thus enabling time-efficient C–N and C–S couplings to be formed quantitatively in 15 h (Figure 8). For comparison, one of the most effective homogeneous catalytic systems comprising CuI together with l-proline as a ligand provided a yield of 66% for the same reaction in a sequential manner (at 90 °C for 24 h for the C–S coupling, then at 110 °C for 48 h for the C–N coupling) with a catalyst loading of 10 mol % and a base loading of 5 equiv.^23^ Thus, it can be concluded that CuBi_2_O_2_CO_3_ catalyzed the cyclization in a concerted manner under mild conditions.^60−66^

Optimization of the reaction time provided a useful tool to demonstrate the advantage of the immobilization process for catalytic purposes (Figure 8 and Table 1). The concentration versus time functions were of saturation curves in all cases. The linear initial parts were suitable for determining the initial turnover frequency (TOF) values.^67^ Both the selectivities and the activities of the catalysts decreased in the order of CuBi_2_O_2_CO_3_ > CuO@Bi_2_O_2_CO_3_ > CuO > Bi_2_O_2_CO_3_. Selectivities did not vary in the course of the reaction. The selectivity over the pristine bismutite (75%) fell far from the level (>95%) achieved with all the other copper-containing composites. It was surprising to observe that the pure CuO was not capable of catalyzing the cyclization reaction, but it could promote S-arylation selectively. This finding indicates that Bi(III) centers are necessary for promoting N-arylation. Although it would have been obvious, the variations in the catalytic activities could not be explained only on the basis of the differences in the specific surface area or pore width distributions (Table S3). The CuO@Bi_2_O_2_CO_3_ composite had the largest surface area and porosity, however, medium activity compared to CuBi_2_O_2_CO_3_. Generally, all of the presented catalysts had a relatively low specific surface area (11.0–21.8 m^2^/g) with very low total pore volumes. All the as-prepared catalysts could be classified into microporous materials according to the IUPAC classification.

**Table 1:** Catalytic Activities and Product Yields Over 5 mol % Catalysts in the Phenothiazine Formation Reaction in a Mixture of DMSO to Water 1:2, Using 2.5 equiv of Bases at 90 °C

	initial TOF (1/h)	phenothiazine yield (mol %)^a^	2-iodoaniline conversion (mol %)^a^	phenothiazine selectivity (%)^a^	dibenzothiophene selectivity (%)^a^
Bi_2_O_2_CO_3_	1.3 ± 0.1	12 ± 1.1	16 ± 1.3	75 ± 1.5	25 ± 0.8
CuO	2.6 ± 0.3	0	24 ± 2.0	0^b^	2 ± 0.5
CuO@Bi_2_O_2_CO_3_	2.5 ± 0.1	47 ± 3.2	48 ± 2.7	98 ± 2.1	2 ± 0.3
CuBi_2_O_2_CO_3_	10.4 ± 0.5	99 ± 1.2	100 ± 1.8	99 ± 1.7	1 ± 0.3

a After 15 h.

b Only C–S coupling reaction occurred.

In addition, the highest yields attained (Table 1, column 3) also differed significantly from each other indicating that the local structure of copper/bismuth ions is a crucial factor. It is revealed that the bimetallic, bismuth–copper systems are more efficient than the single metallic ones. However, the framework-modified bismutite due to the fixed Cu(II) ions in the layered structure exhibited the best catalytic indicators. As far as initial TOF values are concerned (Table 1, column 2), they were almost the same for CuO and CuO@Bi_2_O_2_CO_3_ because the active sites and Cu(II) ions over both materials and their dispersion are low. However, the initial TOF value over CuBi_2_O_2_CO_3_ was much higher than over the others, probably due to the close atomic dispersion, consequently, to the better availability of the Cu(II) sites. So much so, that considering the above detailed, as-prepared copper-loaded bismutite functioned as a Lewis acid/redox cooperative catalyst.

In order to verify that the reactions are catalyzed by the solid substance, a hot filtration test^68^ was performed and the catalytic capability of the obtained solution was determined (Figure S7). The heterocyclization was carried out under the optimized reaction conditions. The bulk catalyst was readily removed by a simple filtration after 4 h. Then, in the absence of CuBi_2_O_2_CO_3_, the conversion of 2-iodoaniline remained at the same level throughout the whole process as it was before the hot filtration. Because the filtrate was catalytically inactive, and there were no leached metal ions verified by ICP-MS measurements, it can be safely stated that leaching did not occur, and the transformation was heterogeneously catalyzed.

**Figure 9:**
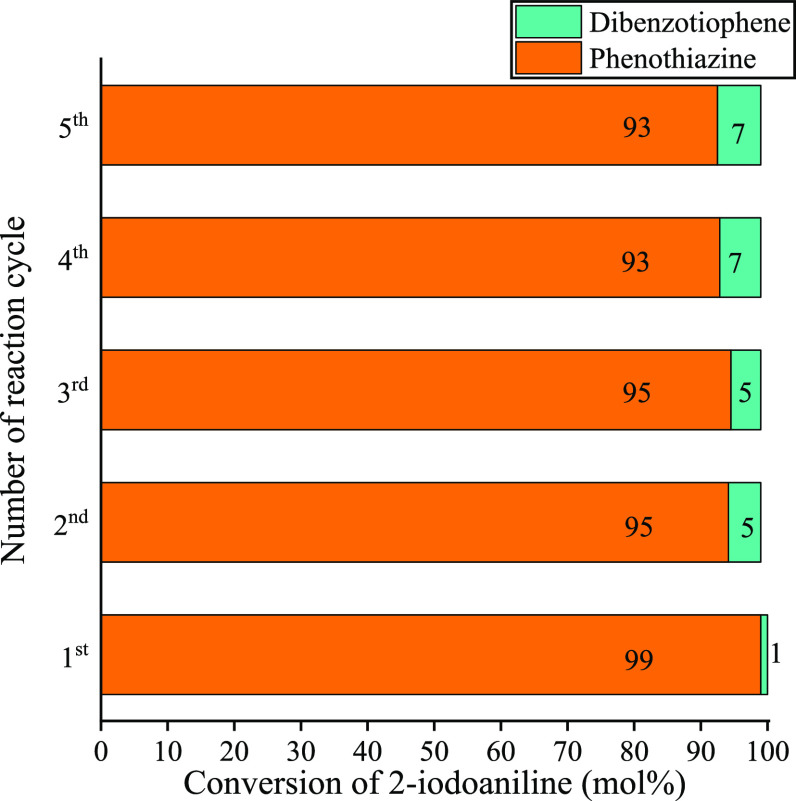
Reusability of CuBi_2_O_2_CO_3_ in the heterocyclization reaction. Reaction conditions: 1 equiv (0.25 M) of 2-iodoaniline, 1.1 equiv of 2-bromobenzenethiol, solvent: DMSO/water 1:2, 2.5 equiv of K_2_CO_3_, 5 mol % CuBi_2_O_2_CO_3_ as the catalyst, 90 °C, 15 h.

After the reaction, the active catalyst was separated from the reaction mixture and reused under identical reaction conditions to establish its recycling characteristics (Figure 9). No remarkable loss in the phenothiazine yield was detected in up to five cycles. Furthermore, through the catalytic runs, the bulk catalyst maintained its stability against leaching, which was confirmed by ICP-MS measurements. In addition, the layered Sillen-like structure did not suffer any substantial degradation shown by *ex situ* XRD analysis performed on the catalyst after the fifth run (Figure S8). The distribution of metal ions and the plate-like morphology of copper bismutite remained nearly unchanged as verified by TEM–EDS measurements (Figure S9). Some impurities appeared on the surface of the nanoplates after the repeated runs, which could be associated with organic deposition accumulated during the catalytic cycles. The increase of the carbon content also supported this as determined by EDX (Figure S9). Furthermore, the *ex situ* XPS study revealed that the surface composition of the reused catalyst after the fifth round was largely unaltered relative to the initial state (Figure S10). However, a fraction (18.4 at. %) of Cu(II) is reduced to metallic copper on the surface after reactions that may also explain the slight decrease in the selectivities experienced. To the best of our knowledge, this is the first reusable catalyst for the synthesis of phenothiazine *via* concerted C–N/C–S heterocyclization.

With the well-designed catalyst and the optimized reaction conditions in hand, the scope of viable derivatives of 2-iodoaniline was probed (Table 2). The obtained high yields and selectivities strengthened the fact of substrate tolerance of the bulk catalyst toward electron-withdrawing as well as electron-donating groups. It has to be mentioned that a commensurate decrease in both the obtained yields and selectivities could be seen in the presence of the electron-donating groups. Besides, it has been also found that there was no alternative starting material for substituting 2-iodoaniline to achieve efficient heterocyclization. Here, the time-effective catalyst provided such a high efficiency toward heterocyclization of all presented raw materials that is higher than ever to experience.

**Table 2:**
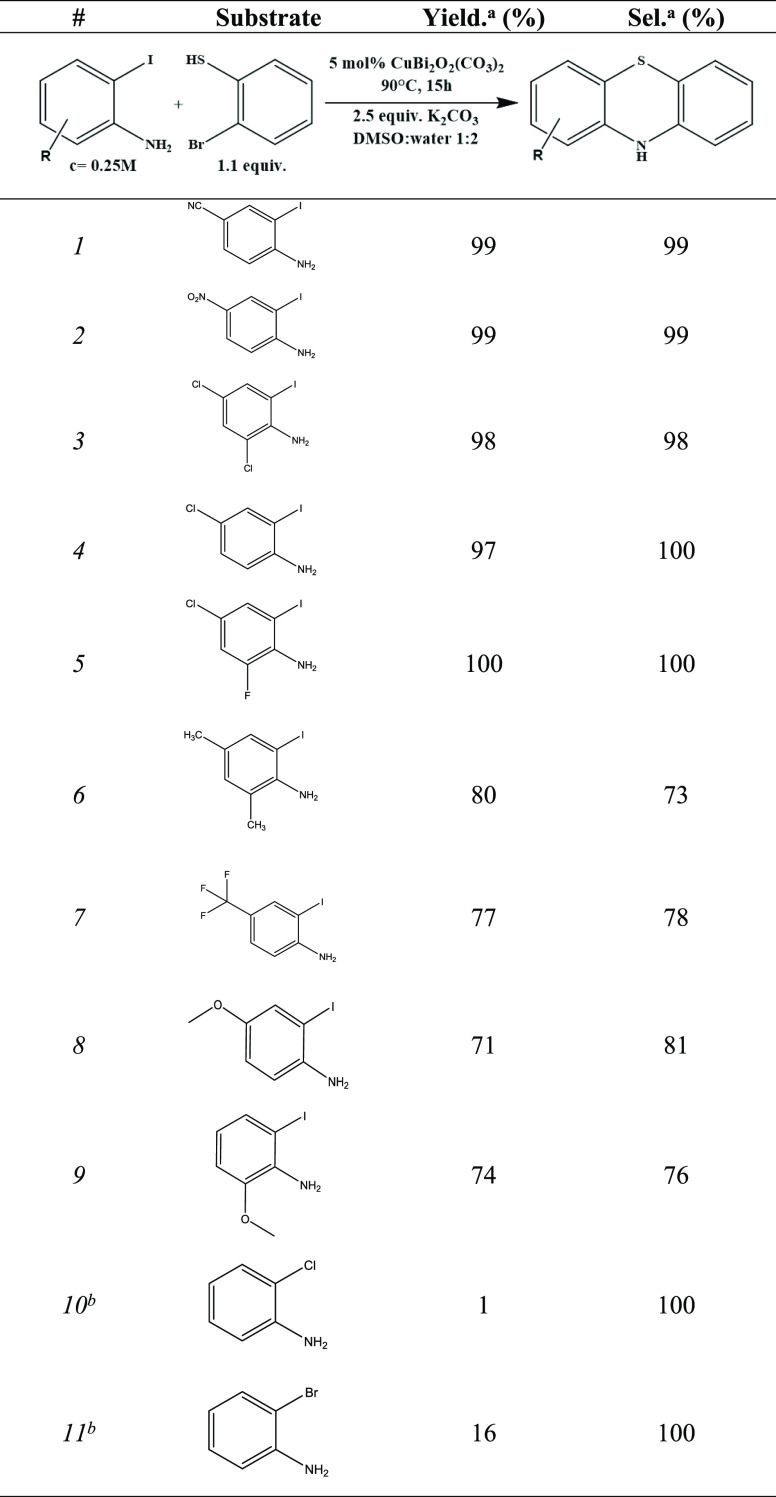
Scope of Phenothiazine Formation*via* Concerted C–S and C–N Couplings

a Determined by GC–MS analysis of the crude product.

b *T* = 110 °C.

## 4 Conclusions

Herein, a well-designed heterogeneous bifunctional catalyst has been reported to promote synthetically useful heterocyclization involving N- and S-arylations with remarkable efficiency. By immobilization within the bismutite (Sillen-type) framework as a suitable host, copper(II) ions could occupy stable interlamellar positions as justified by a large series of analytical techniques. Similarly to other intercalated cations previously reported [e.g., Ag(I) and Ca(II)], copper ions were surrounded by hydroxyl and carbonate groups, which led to the formation of malachite-like complex anions being dispersed among the layers, as identified by various spectroscopic methods. As a result of efficient catalytic heterocyclization, phenothiazine and its substituted derivatives were successfully synthesized under mild reaction conditions and within short reaction times. Importantly, this reaction has been achieved under heterogeneous catalytic conditions for the first time. Moreover, C–S and C–N coupling reactions resulting in concomitant heterocyclization took place simultaneously due to the fact that the Sillen-like structure operated efficiently in a cooperative manner ensuring Lewis centers, especially, bismuth(III) centers and catalytic copper(II) ions with variable charges demonstrably. Additionally, the copper-bismutite system presented outstanding resistance against leaching and exhibited high degree of recyclability without the loss of activity or selectivity.

## Supporting Information Available

Applied chemicals; details of the calculation methods; calculated cell parameters; TG analysis data; XRD patterns of the heat-treated product; TEM/TEM–EDX pictures of the as-prepared CuBi_2_O_2_CO_3_; XPS survey scans of the bismutites; conversion and selectivity data of the catalytic optimization procedure; hot filtration test of CuBi_2_O_2_CO_3_; XRD and TEM/TEM–EDX as well as XPS characterization of the used CuBi_2_O_2_CO_3_; and ^1^H and ^13^C NMR spectral data of all the produced phenothiazine derivatives (PDF)

## References

[ref1] Matsuo K.; Yasuda T. Blue Thermally Activated Delayed Fluorescence Emitters Incorporating Acridan Analogues with Heavy Group 14 Elements for High-Efficiency Doped and Non-Doped OLEDs. Chem. Sci. 2019, 10, 10687–10697. 10.1039/c9sc04492b. 32206251 PMC7069243

[ref2] Li W.; Li B.; Cai X.; Gan L.; Xu Z.; Li W.; Liu K.; Chen D.; Su S. J. Tri-Spiral Donor for High Efficiency and Versatile Blue Thermally Activated Delayed Fluorescence Materials. Angew. Chem., Int. Ed. 2019, 58, 11301–11305. 10.1002/anie.201904272. 31192492

[ref3] Xu S.; Liu T.; Mu Y.; Wang Y.-F.; Chi Z.; Lo C.-C.; Liu S.; Zhang Y.; Lien A.; Xu J. An Organic Molecule with Asymmetric Structure Exhibiting Aggregation-Induced Emission, Delayed Fluorescence, and Mechanoluminescence. Angew. Chem., Int. Ed. 2015, 127, 888–892. 10.1002/anie.201409767. 25469742

[ref4] Rhee H.-W.; Choi S. J.; Yoo S. H.; Jang Y. O.; Park H. H.; Pinto R. M.; Cameselle J. C.; Sandoval F. J.; Roje S.; Han K.; Chung D. S.; Suh J.; Hong J.-I. A Bifunctional Molecule as an Artificial Flavin Mononucleotide Cyclase and a Chemosensor for Selective Fluorescent Detection of Flavins. J. Am. Chem. Soc. 2009, 131, 10107–10112. 10.1021/ja9018012. 19569646

[ref5] Mondal S.; Samim Ali S.; Manna S.; Maiti K.; Uddin M. R.; Mandal S.; Mandal D.; Mahapatra A. K. A Benzopyrylium-Phenothiazine Conjugate of a Flavylium Derivative as a Fluorescent Chemosensor for Cyanide in Aqueous Media and Its Bioimaging. New J. Chem. 2017, 41, 12581–12588. 10.1039/c7nj02716h.

[ref6] El-Shishtawy R. M.; Al-Zahrani F. A. M.; Al-amshany Z. M.; Asiri A. M. Synthesis of a New Fluorescent Cyanide Chemosensor Based on Phenothiazine Derivative. Sens. Actuators, B 2017, 240, 288–296. 10.1016/j.snb.2016.08.168.

[ref7] Weiss E. A.; Tauber M. J.; Kelley R. F.; Ahrens M. J.; Ratner M. A.; Wasielewski M. R. Conformationally Gated Switching between Superexchange and Hopping within Oligo- p -Phenylene-Based Molecular Wires. J. Am. Chem. Soc. 2005, 127, 11842–11850. 10.1021/ja052901j. 16104763

[ref8] Hua T.; Huang Z.-S.; Cai K.; Wang L.; Tang H.; Meier H.; Cao D. Phenothiazine Dye Featuring Encapsulated Insulated Molecular Wire as Auxiliary Donor for High Photovoltage of Dye-Sensitized Solar Cells by Suppression of Aggregation. Electrochim. Acta 2019, 302, 225–233. 10.1016/j.electacta.2019.02.013.

[ref9] Woods S. W. Chlorpromazine Equivalent Doses for the Newer Atypical Antipsychotics. J. Clin. Psychiatry 2003, 64, 663–667. 10.4088/JCP.v64n0607. 12823080

[ref10] Warman A. J.; Rito T. S.; Fisher N. E.; Moss D. M.; Berry N. G.; O’Neill P. M.; Ward S. A.; Biagini G. A. Antitubercular Pharmacodynamics of Phenothiazines. J. Antimicrob. Chemother. 2013, 68, 869–880. 10.1093/jac/dks483. 23228936 PMC3594496

[ref11] Montoya M. C.; DiDone L.; Heier R. F.; Meyers M. J.; Krysan D. J. Antifungal Phenothiazines: Optimization, Characterization of Mechanism, and Modulation of Neuroreceptor Activity. ACS Infect. Dis. 2018, 4, 499–507. 10.1021/acsinfecdis.7b00157. 29058407 PMC8994671

[ref12] Xu M.; Peng Y.; Zhu L.; Wang S.; Ji J.; Rakesh K. P. Triazole Derivatives as Inhibitors of Alzheimer’s Disease: Current Developments and Structure-Activity Relationships. Eur. J. Med. Chem. 2019, 180, 656–672. 10.1016/j.ejmech.2019.07.059. 31352246

[ref13] Darvesh S.; Darvesh K. V.; McDonald R. S.; Mataija D.; Walsh R.; Mothana S.; Lockridge O.; Martin E. Carbamates with Differential Mechanism of Inhibition Toward Acetylcholinesterase and Butyrylcholinesterase. J. Med. Chem. 2008, 51, 4200–4212. 10.1021/jm8002075. 18570368

[ref14] Bisi A.; Meli M.; Gobbi S.; Rampa A.; Tolomeo M.; Dusonchet L. Multidrug Resistance Reverting Activity and Antitumor Profile of New Phenothiazine Derivatives. Bioorg. Med. Chem. 2008, 16, 6474–6482. 10.1016/j.bmc.2008.05.040. 18522868

[ref15] Liu X.; Wang X.-J. Potential Inhibitors against 2019-NCoV Coronavirus M Protease from Clinically Approved Medicines. J. Genet. Genomics 2020, 47, 119–121. 10.1016/j.jgg.2020.02.001. 32173287 PMC7128649

[ref16] Mao L.; Wu Y.; Jiang J.; Guo X.; Heng P.; Wang L.; Zhang J. Rational Design of Phenothiazine-Based Organic Dyes for Dye-Sensitized Solar Cells: The Influence of π-Spacers and Intermolecular Aggregation on Their Photovoltaic Performances. J. Phys. Chem. C 2020, 124, 9233–9242. 10.1021/acs.jpcc.0c01875.

[ref17] Boota M.; Bécuwe M.; Gogotsi Y. Phenothiazine-MXene Aqueous Asymmetric Pseudocapacitors. ACS Appl. Energy Mater. 2020, 3, 3144–3149. 10.1021/acsaem.9b02404.

[ref18] Nakatani Y.; Shimaki Y.; Dutta D.; Muench S. P.; Ireton K.; Cook G. M.; Jeuken L. J. C. Unprecedented Properties of Phenothiazines Unraveled by a NDH-2 Bioelectrochemical Assay Platform. J. Am. Chem. Soc. 2020, 142, 1311–1320. 10.1021/jacs.9b10254. 31880924

[ref19] Mayer M.; Lang P. T.; Gerber S.; Madrid P. B.; Pinto I. G.; Guy R. K.; James T. L. Synthesis and Testing of a Focused Phenothiazine Library for Binding to HIV-1 TAR RNA. Chem. Biol. 2006, 13, 993–1000. 10.1016/j.chembiol.2006.07.009. 16984889

[ref20] Sharma N.; Gupta R.; Kumar M.; Gupta R. R. Synthesis of Fluorophenothiazines via Smiles Rearrangement and Their Conversion into Sulfones. J. Fluorine Chem. 1999, 98, 153–157. 10.1016/S0022-1139(99)00098-6.

[ref21] Jepsen T. H.; Larsen M.; Jørgensen M.; Nielsen M. B. Three-Step Synthesis of (Thio)Xanthene and Dibenzothiepine/Dibenzoxepine by an Intramolecular Mizoroki-Heck Reaction of Diaryl (Thio)Ethers. Synlett 2012, 23, 418–422. 10.1055/s-0031-1290317.

[ref22] Dai C.; Sun X.; Tu X.; Wu L.; Zhan D.; Zeng Q. Synthesis of Phenothiazines via Ligand-Free CuI-Catalyzed Cascade C-S and C-N Coupling of Aryl Ortho-Dihalides and Ortho-Aminobenzenethiols. Chem. Commun. 2012, 48, 5367–5369. 10.1039/c2cc30814b. 22525793

[ref23] Ma D.; Geng Q.; Zhang H.; Jiang Y. Assembly of Substituted Phenothiazines by a Sequentially Controlled CuI/L-Proline-Catalyzed Cascade C-S and C-N Bond Formation. Angew. Chem., Int. Ed. Engl 2010, 49, 1291–1294. 10.1002/anie.200905646. 20058286

[ref24] Huang M.; Hou J.; Yang R.; Zhang L.; Zhu X.; Wan Y. A Catalyst System, Copper/ N -Methoxy-1 H -Pyrrole-2-Carboxamide, for the Synthesis of Phenothiazines in Poly(Ethylene Glycol). Synthesis 2014, 46, 3356–3364. 10.1055/s-0034-1379045.

[ref25] Dhakshinamoorthy A.; Asiri A. M.; Garcia H. Formation of C-C and C-Heteroatom Bonds by C-H Activation by Metal Organic Frameworks as Catalysts or Supports. ACS Catal. 2019, 9, 1081–1102. 10.1021/acscatal.8b04506.

[ref26] Zhu C.; Schwarz J. L.; Cembellín S.; Greßies S.; Glorius F. Highly Selective Manganese(I)/Lewis Acid Cocatalyzed Direct C–H Propargylation Using Bromoallenes. Angew. Chem., Int. Ed. 2018, 57, 437–441. 10.1002/anie.201710835. 29141113

[ref27] Chang X.; Zhang J.; Peng L.; Guo C. Collective Synthesis of Acetylenic Pharmaceuticals via Enantioselective Nickel/Lewis Acid-Catalyzed Propargylic Alkylation. Nat. Commun. 2021, 12, 1–9. 10.1038/s41467-020-20644-9. 33436637 PMC7803749

[ref28] Sako M.; Takeuchi Y.; Tsujihara T.; Kodera J.; Kawano T.; Takizawa S.; Sasai H. Efficient Enantioselective Synthesis of Oxahelicenes Using Redox/Acid Cooperative Catalysts. J. Am. Chem. Soc. 2016, 138, 11481–11484. 10.1021/jacs.6b07424. 27574874

[ref29] Zhou B.; Hu Y.; Wang C. Manganese-Catalyzed Direct Nucleophilic C(Sp^2^)-H Addition to Aldehydes and Nitriles. Angew. Chem., Int. Ed. 2015, 54, 13659–13663. 10.1002/anie.201506187. 26360929

[ref30] Monnier F.; Taillefer M. Catalytic C-C, C-N, and C-O Ullmann-Type Coupling Reactions. Angew. Chem., Int. Ed. 2009, 48, 6954–6971. 10.1002/anie.200804497. 19681081

[ref31] Kelly S. M.; Han C.; Tung L.; Gosselin F. Chemoselective Copper-Catalyzed Ullmann-Type Coupling of Oxazolidinones with Bromoiodoarenes. Org. Lett. 2017, 19, 3021–3024. 10.1021/acs.orglett.7b01304. 28530104

[ref32] Cao Q.; Peng H. Y.; Cheng Y.; Dong Z. B. A Highly Efficient CuCl_2_ -Catalyzed C-S Coupling of Aryl Iodides with Tetraalkylthiuram Disulfides: Synthesis of Aryl Dithiocarbamates. Synthesis 2018, 50, 1527–1534. 10.1055/s-0036-1589166.

[ref33] Ötvös S. B.; Pálinkó I.; Fülöp F. Catalytic Use of Layered Materials for Fine Chemical Syntheses. Catal. Sci. Technol. 2019, 9, 47–60. 10.1039/c8cy02156b.

[ref34] Varga G.; Kocsis M.; Kukovecz Á.; Kónya Z.; Djerdj I.; Sipos P.; Pálinkó I. CuIBiOI Is an Efficient Novel Catalyst in Ullmann-Type CN Couplings with Wide Scope—A Rare Non-Photocatalytic Application. Mol. Catal. 2020, 493, 111072 10.1016/j.mcat.2020.111072.

[ref35] Ötvös S. B.; Mészáros R.; Varga G.; Kocsis M.; Kónya Z.; Kukovecz Á.; Pusztai P.; Sipos P.; Pálinkó I.; Fülöp F. A Mineralogically-Inspired Silver-Bismuth Hybrid Material: An Efficient Heterogeneous Catalyst for the Direct Synthesis of Nitriles from Terminal Alkynes. Green Chem. 2018, 20, 1007–1019. 10.1039/c7gc02487h.

[ref36] Mészáros R.; Ötvös S. B.; Varga G.; Böszörményi É.; Kocsis M.; Karádi K.; Kónya Z.; Kukovecz Á.; Pálinkó I.; Fülöp F. A Mineralogically-Inspired Silver–Bismuth Hybrid Material: Structure, Stability and Application for Catalytic Benzyl Alcohol Dehydrogenations under Continuous Flow Conditions. Mol. Catal. 2020, 498, 111263 10.1016/j.mcat.2020.111263.

[ref37] Liu A.; Liu L.; Cao Y.; Wang J.; Si R.; Gao F.; Dong L. Controlling Dynamic Structural Transformation of Atomically Dispersed CuOx Species and Influence on Their Catalytic Performances. ACS Catal. 2019, 9, 9840–9851. 10.1021/acscatal.9b02773.

[ref38] Lábár J. L.; Adamik M.; Barna B. P.; Czigány Z.; Fogarassy Z.; Horváth Z. E.; Geszti O.; Misják F.; Morgiel J.; Radnóczi G.; Sáfrán G.; Székely L.; Szüts T. Electron Diffraction Based Analysis of Phase Fractions and Texture in Nanocrystalline Thin Films, Part III: Application Examples. Microsc. Microanal. 2012, 18, 406–420. 10.1017/S1431927611012803. 22436336

[ref39] Zhang Y.; Zhang X.; Ling Y.; Li F.; Bond A. M.; Zhang J. Controllable Synthesis of Few-Layer Bismuth Subcarbonate by Electrochemical Exfoliation for Enhanced CO_2_ Reduction Performance. Angew. Chem. 2018, 130, 13467–13471. 10.1002/ange.201807466. 30129234

[ref40] Huang H.; Li X.; Wang J.; Dong F.; Chu P. K.; Zhang T.; Zhang Y. Anionic Group Self-Doping as a Promising Strategy: Band-Gap Engineering and Multi-Functional Applications of High-Performance CO_3_^2-^-Doped Bi_2_O_2_CO_3_. ACS Catal. 2015, 5, 4094–4103. 10.1021/acscatal.5b00444.

[ref41] Naatz H.; Lin S.; Li R.; Jiang W.; Ji Z.; Chang C. H.; Köser J.; Thöming J.; Xia T.; Nel A. E.; Mädler L.; Pokhrel S. Safe-by-Design CuO Nanoparticles via Fe-Doping, Cu-O Bond Length Variation, and Biological Assessment in Cells and Zebrafish Embryos. ACS Nano 2017, 11, 501–515. 10.1021/acsnano.6b06495. 28026936 PMC5824973

[ref42] Malik V.; Uma S. Effective Catalytic Reduction of Aromatic Nitrocompounds Using Mineral Beyerite, CaBi_2_O_2_(CO_3_)_2_. J. Environ. Chem. Eng. 2018, 6, 4755–4763. 10.1016/j.jece.2018.06.068.

[ref43] Selvamani T.; Gnana Sundara Raj B.; Anandan S.; Wu J. J.; Ashokkumar M. Synthesis of Morphology-Controlled Bismutite for Selective Applications. Phys. Chem. Chem. Phys. 2016, 18, 7768–7779. 10.1039/c5cp07523h. 26910578

[ref44] Szabados M.; Adél Ádám A.; Traj P.; Muráth S.; Baán K.; Bélteky P.; Kónya Z.; Kukovecz Á.; Sipos P.; Pálinkó I. Mechanochemical and Wet Chemical Syntheses of CaIn-Layered Double Hydroxide and Its Performance in a Transesterification Reaction Compared to Those of Other Ca_2_M(III) Hydrocalumites (M: Al, Sc, V, Cr, Fe, Ga) and Mg(II)-, Ni(II)-, Co(II)- or Zn(II)-Based hydrotalcites. J. Catal. 2020, 391, 282–297. 10.1016/j.jcat.2020.07.038.

[ref45] Frost R. L.; Ding Z.; Kloprogge J. T.; Martens W. N. Thermal Stability of Azurite and Malachite in Relation to the Formation of Mediaeval Glass and Glazes. Thermochim. Acta 2002, 390, 133–144. 10.1016/S0040-6031(02)00127-2.

[ref46] Tsang C.-F.; Meen J. K.; Elthon D. Phase Equilibria of the Bismuth Oxide-Copper Oxide System in Oxygen at 1 Atm. J. Am. Ceram. Soc. 1994, 77, 3119–3124. 10.1111/j.1151-2916.1994.tb04558.x.

[ref47] Taylor P.; Sunder S.; Lopata V. J. Structure, Spectra, and Stability of Solid Bismuth Carbonates. Can. J. Chem. 1984, 62, 2863–2873. 10.1139/v84-484.

[ref48] Tobon-Zapata G. E.; Etcheverry S. B.; Baran E. J. Vibrational Spectrum of Bismuth Subcarbonate. J. Mater. Sci. Lett. 1997, 16, 656–657. 10.1023/A:1018527602604.

[ref49] Frost R. L.; Martens W. N.; Rintoul L.; Mahmutagic E.; Kloprogge J. T. Raman Spectroscopic Study of Azurite and Malachite at 298 and 77 K. J. Raman Spectrosc. 2002, 33, 252–259. 10.1002/jrs.848.

[ref50] Xu C.; Qiu P.; Li L.; Chen H.; Jiang F.; Wang X. Bismuth Subcarbonate with Designer Defects for Broad-Spectrum Photocatalytic Nitrogen Fixation. ACS Appl. Mater. Interfaces 2018, 10, 25321–25328. 10.1021/acsami.8b05925. 29969006

[ref51] Dong F.; Lee S. C.; Wu Z.; Huang Y.; Fu M.; Ho W.-K.; Zou S.; Wang B. Rose-like Monodisperse Bismuth Subcarbonate Hierarchical Hollow Microspheres: One-Pot Template-Free Fabrication and Excellent Visible Light Photocatalytic Activity and Photochemical Stability for NO Removal in Indoor Air. J. Hazard. Mater. 2011, 195, 346–354. 10.1016/j.jhazmat.2011.08.050. 21903327

[ref52] Coenen K.; Gallucci F.; Mezari B.; Hensen E.; van Sint Annaland M. An In-Situ IR Study on the Adsorption of CO_2_ and H_2_O on Hydrotalcites. J. CO_2_ Util. 2018, 24, 228–239. 10.1016/j.jcou.2018.01.008.

[ref53] Stoilova D.; Koleva V.; Vassileva V. Infrared Study of Some Synthetic Phases of Malachite (Cu_2_(OH)_2_CO_3_)-Hydrozincite (Zn_5_(OH)_6_(CO_3_)_2_) Series. Spectrochim. Acta, Part A 2002, 58, 2051–2059. 10.1016/S1386-1425(01)00677-1. 12164502

[ref54] Frost R. L.; Reddy B. J.; Wain D. L.; Martens W. N. Identification of the Rosasite Group Minerals-An Application of near Infrared Spectroscopy. Spectrochim. Acta, Part A 2007, 66, 1075–1081. 10.1016/j.saa.2006.04.043. 17027326

[ref55] Dong F.; Sun Y.; Ho W.-K.; Wu Z. Controlled Synthesis, Growth Mechanism and Highly Efficient Solar Photocatalysis of Nitrogen-Doped Bismuth Subcarbonate Hierarchical Nanosheets Architectures. Dalton Trans. 2012, 41, 8270–8284. 10.1039/c2dt30570d. 22622668

[ref56] Huai Y.; Qian Y.; Peng Y. Re-Evaluating the Sulphidisation Reaction on Malachite Surface through Electrochemical and Cryo XPS Studies. Appl. Surf. Sci. 2020, 531, 147334 10.1016/j.apsusc.2020.147334.

[ref57] Bothwell J. M.; Krabbe S. W.; Mohan R. S. Applications of Bismuth(III) Compounds in Organic Synthesis. Chem. Soc. Rev. 2011, 40, 4649–4707. 10.1039/c0cs00206b. 21589974

[ref58] Ritschel B.; Lichtenberg C. Cationic Bismuth Compounds in Organic Synthesis and Catalysis: New Prospects for CH Activation. Synlett 2018, 29, 2213–2217. 10.1055/s-0037-1610160.

[ref59] Prat D.; Wells A.; Hayler J.; Sneddon H.; McElroy C. R.; Abou-Shehada S.; Dunn P. J. CHEM21 Selection Guide of Classical- and Less Classical-Solvents. Green Chem. 2016, 18, 288–296. 10.1039/C5GC01008J.

[ref60] Fan M.; Zhou W.; Jiang Y.; Ma D. Assembly of Primary (Hetero)Arylamines via CuI/Oxalic Diamide-Catalyzed Coupling of Aryl Chlorides and Ammonia. Org. Lett. 2015, 17, 5934–5937. 10.1021/acs.orglett.5b03230. 26600015

[ref61] Garnier T.; Danel M.; Magné V.; Pujol A.; Bénéteau V.; Pale P.; Chassaing S. Copper(I)–USY as a Ligand-Free and Recyclable Catalyst for Ullmann-Type O -, N -, S -, and C -Arylation Reactions: Scope and Application to Total Synthesis. J. Org. Chem. 2018, 83, 6408–6422. 10.1021/acs.joc.8b00620. 29790337

[ref62] Mantovani A. C.; Goulart T. A. C.; Back D. F.; Zeni G. Synthesis of Pyridazinones through the Copper(I)-Catalyzed Multicomponent Reaction of Aldehydes, Hydrazines, and Alkynylesters. Chem.—Eur. J. 2014, 20, 12663–12668. 10.1002/chem.201403873. 25124722

[ref63] Wang L.; Zhang J.; Sun J.; Zhu L.; Zhang H.; Liu F.; Zheng D.; Meng X.; Shi X.; Xiao F.-S. Copper-Incorporated Porous Polydivinylbenzene as Efficient and Recyclable Heterogeneous Catalyst in Ullmann Biaryl Ether Coupling. ChemCatChem 2013, 5, 1606–1613. 10.1002/cctc.201200578.

[ref64] Li M.; Xing X.; Ma Z.; Lv J.; Fu P.; Li Z. Synthesis of Composition Tunable and (111)-Faceted Cu/Cu_2_O Nanoparticles toward Photocatalytic, Ligand-Free, and Solvent-Free C-N Ullmann Coupling Reactions. ACS Sustainable Chem. Eng. 2018, 6, 5495–5503. 10.1021/acssuschemeng.8b00350.

[ref65] Orha L.; Tukacs J. M.; Gyarmati B.; Szilágyi A.; Kollár L.; Mika L. T. Modular Synthesis of γ-Valerolactone-Based Ionic Liquids and Their Application as Alternative Media for Copper-Catalyzed Ullmann-Type Coupling Reactions. ACS Sustainable Chem. Eng. 2018, 6, 5097–5104. 10.1021/acssuschemeng.7b04775.

[ref66] Rovira M.; Soler M.; Güell I.; Wang M.-Z.; Gómez L.; Ribas X. Orthogonal Discrimination among Functional Groups in Ullmann-Type C-O and C-N Couplings. J. Org. Chem. 2016, 81, 7315–7325. 10.1021/acs.joc.6b01035. 27249644

[ref67] Kozuch S.; Martin J. M. L. “Turning over” Definitions in Catalytic Cycles. ACS Catal. 2012, 2, 2787–2794. 10.1021/cs3005264.

[ref68] Ventura-Espinosa D.; Martín S.; Mata J. A. The Non-Innocent Role of Graphene in the Formation/Immobilization of Ultra-Small Gold Nanoparticles Functionalized with N-Heterocyclic Carbene Ligands. J. Catal. 2019, 375, 419–426. 10.1016/j.jcat.2019.06.009.

