# Supplementary material for: Copper-Loaded Layered Bismuth Subcarbonate—Efficient Multifunctional Heterogeneous Catalyst for Concerted C–S/C–N Heterocyclization
Source: ACS Appl Mater Interfaces. 2021 Sep 3;13(36):42650–61. doi: 10.1021/acsami.1c09234 (PMC8447192; doi:10.1021/acsami.1c09234)
Supplement: Supplementary file 1 — am1c09234_si_001.pdf [file am1c09234_si_001.pdf]

Supporting Information

Copper-loaded layered bismuth subcarbonate – efficient multifunctional heterogeneous catalyst for concerted C–S/C–N heterocyclization

*Marianna Kocsis[a,b], Sándor B. Ötvös[c], Gergely F. Samu[d], Zsolt Fogarassy[e], Béla Pécz[e], Ákos Kukovecz[f], Zoltán Kónya[f,g], Pál Sipos[b,h], †István Pálinkó[a,b] and Gábor Varga[b,i]**

[a]Department of Organic Chemistry, University of Szeged, Dóm tér 8, Szeged, H-6720 Hungary
[b]Materials and Solution Structure Research Group, and Interdisciplinary Excellence Centre, Institute of Chemistry, University of Szeged, Aradi Vértanúk tere 1, Szeged, H-6720 Hungary
[c]Institute of Chemistry, University of Graz, NAWI Graz, Heinrichstrasse 28, Graz, A-8010 Austria.
[d]Department of Physical Chemistry and Materials Science, Interdisciplinary Excellence Centre, University of Szeged, Szeged H-6720, Hungary
[e]Institute of Technical Physics and Materials Sciences, Centre for Energy Research, H.A.S., H-1121 Budapest, Konkoly Thege M. út 29-33, Hungary
[f]Department of Applied and Environmental Chemistry, University of Szeged, Rerrich Béla tér 1, Szeged, H-6720 Hungary
[g]MTA-SZTE Reaction Kinetics and Surface Chemistry Research Group, Rerrich Béla tér 1, Szeged, H-6720 Hungary
[h]Department of Inorganic and Analytical Chemistry, University of Szeged, Dóm tér 7, Szeged, H-6720 Hungary
[i]Department of Physical Chemistry and Materials Science, University of Szeged, Rerrich Béla tér 1, Szeged, H-6720 Hungary

S1. Materials

All chemicals used were analytical grade Sigma-Aldrich products, and were applied without further purification.

Copper(II)-oxide (CuO); bismuth(III)nitrate-pentahydrate ($Bi(NO_3)_3 \times 5H_2O$); copper(II)nitrate-trihydrate ($Cu(NO_3) \times 3H_2O$); ammonia solution (25%; NH_4OH); sodium carbonate (Na_2CO_3); concentrated nitric acid (cc. HNO_3); potassium carbonate (K_2CO_3); 2-iodoaniline ($IC_6H_4NH_2$); 2-bromothiophenol (BrC_6H_4SH); dimethyl sulfoxide (C_2H_6OS; DMSO); ethyl acetate ($CH_3COOCH_2CH_3$; EtOAc); ethylene glycol ($(CH_2OH)_2$); 5-methyloxolan-2-one (γ-valerolactone, $C_5H_8O_2$); pentane-2,4-dione (acethylacetone, $C_5H_8O_2$); N,N-dimethylacetamide ($CH_3CON(CH_3)_2$; DMA); N,N-dimethylformamide ($HCON(CH_3)_2$; DMF); 2-butanone ($CH_3C(O)CH_2CH_3$; methyl ethyl ketone (MEK)); 4-amino-3-iodobenzonitrile ($IC_6H_3(NH_2)CN$); 2-iodo-4-nitroaniline ($IC_6H_3(NO_2)NH_2$); 4-chloro-2-iodoaniline ($ClC_6H_3(I)NH_2$); 2,4-dichloro-6-iodoaniline ($Cl_2C_6H_3(I)NH_2$); 4-chloro-2-fluoro-6-iodoaniline ($(Cl)(F)C_6H_3(I)NH_2$); 2-iodo-4,6-dimethylaniline ($I(CH_3)_2(C_6H_3)NH_2$).

S2. Applied equations

Bragg's law: The distance of one layer together with the interlayer distance was calculated by Bragg's law:

(1) $$n\lambda = 2d_{hkl}\sin\theta$$

where n is an integer; λ is the wavelength of the incident light, d_{hkl} is the lattice spacing and θ is the angle of incidence.

Scherrer equation: The primer crystallite size was calculated by Sherrer equation:

(2) $$D = (K \times \lambda)/(\beta \times \cos\theta)$$

where D is the average crystallite size, β is the line broadening in radians while θ is the Bragg angle as well as λ is the wavelength of the incident light.

Cell parameters calculation: the corresponding cell parameters were determined by the following equations:

(3) for tetragonal crystal structure: $\frac{1}{d_{hkl}^2} = \left[h^2 + k^2 + l^2 \times \left(\frac{a}{c}\right)^2\right]\frac{1}{a^2}$; $V = a^2 \times c$

(4) for orthorhombic crystal structure: $\frac{1}{d_{hkl}^2} = \frac{h^2}{a^2} + \frac{k^2}{b^2} + \frac{l^2}{c^2}$; $V = a \times b \times c$

where d_{hkl} is the lattice spacing, h/k/l are the corresponding Miller indexes, a/b/c are the corresponding lattice parameters while V is the corresponding cell volume.

Catalytic indicators calculation:

(5) Conversion of 2-iodoaniline (C):

$$C = \frac{n_{(0)\,2\text{-iodoaniline}}}{n_{(0)\,2\text{-iodoaniline}} - n_{(t)\,2\text{-iodoaniline}}} \times 100$$

(6) Selectivity of phenothiazine (S):

$$S = \frac{n_{(t)\,2\text{-iodoaniline}}}{n_{(t)2\text{-iodoaniline}} + n_{(t)\text{dibenzotiophene}} + n_{(t)\text{thianthrene}} + n_{(t)\text{dibromodiphenylsulfide}} + n_{(t)1,2\text{-bis(2-bromophenyl)disulfide}}} \times 100$$

(7) Yield of phenothiazine (Y):

$$Y = S \times C$$

where n_0 is the corresponding initial volume of the reactants/products while n_t is the corresponding volume of the reactants/products at a given time.

S3. Supporting figures and tables

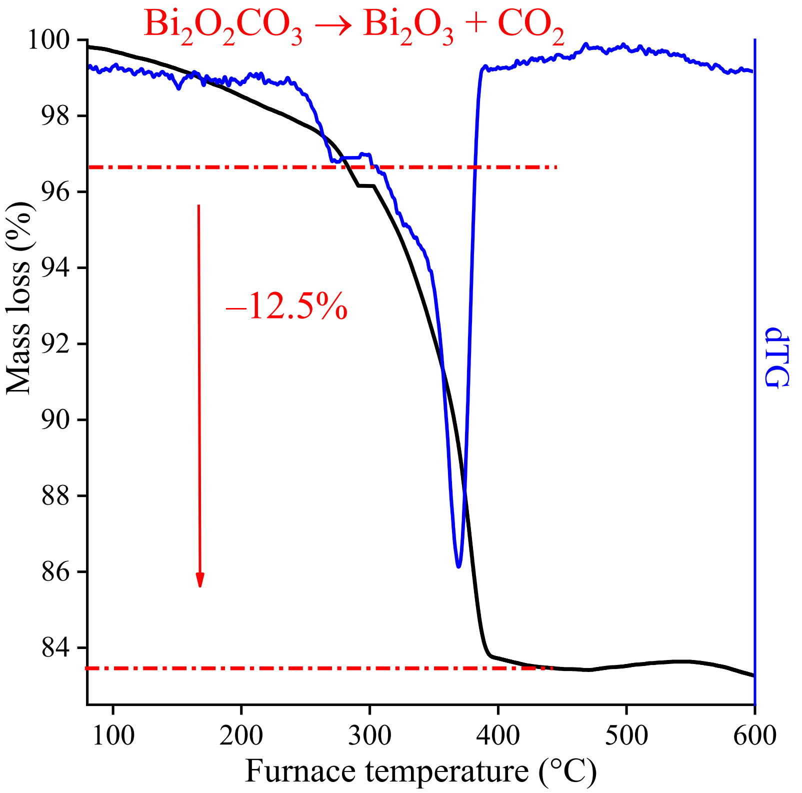

Figure S1. TG/DTG-curves of $Bi_2O_2CO_3$.

Table S1. Calculated cell parameters, interlayer distances and actual molar ratios.

	**d_{002} (nm)[1,2]**	**a (nm)[1]**	**b (nm)[1]**	**c (nm)[1]**	**V (nm^3)[1]**	**D (nm)[1,3]**	**Cu:Bi ratio[4]**
$Bi_2O_2CO_3$[5]	0.791	0.341	—	1.584	0.184	43.15	—
$CuO@Bi_2O_2CO_3$[5]	0.791	0.343	—	1.578	0.186	41.10	0.23
$CuBi_2O_2CO_3$[6]	0.657	0.353	0.343	1.314	0.159	17.26	0.25

[1]based on XRD; [2]by Bragg's law; [3]by Scherrer equation, [4]based on ICP-MS; [5]tetragonal crystal structure; [6]orthorombic crystal structure

Table S2. Results of TG/DTG analysis for as-prepared bismutite structures.

Name	Original formula[1]	Temperature range (°C)	Leaving species	Mass loss (%)	Formula of the obtained solids
$Bi_2O_2CO_3$	$Bi_2O_2(CO_3)_x$× n H_2O	25–300	H_2O	4	$Bi_2O_2(CO_3)_x$
		300–400	CO_3^{2-}	12.5	α–Bi_2O_3[2]
$CuBi_2O_2CO_3$	$Cu_{0.5}Bi_2O_2(CO_3)_{1.25}(OH)_{0.5}$ × n H_2O	25–200	H_2O	2.5	$Cu_{0.5}Bi_2O_2(CO_3)_{1.25}(OH)_{0.5}$
		200–280	H_2O/OH^-	2.8	$Cu_{0.5}Bi_2O_2(CO_3)_{1.25}(OH)_{0.5-x}$
		290–350<br>310–400	OH^-<br>CO_3^{2-}	13	$Cu_2Bi_2O_5$+$Bi_2O_3(CO_3)_x$
		400–600	CO_3^{2-}	1	$Bi_{2+x}Cu_{1-x}O_4$[2]

[1]Actual molar ratio of metal ions was determined by ICP–MS; [2]confirmed by XRD

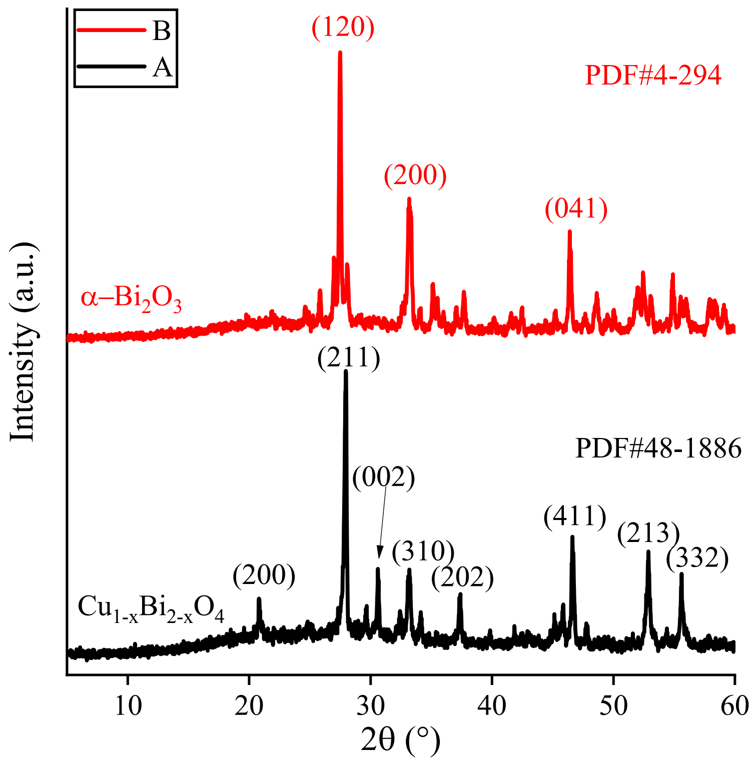

Figure S2. XRD patterns of (A) $CuBi_2O_2CO_3$; (B) $Bi_2O_2CO_3$ after heat treatment of 600 °C.

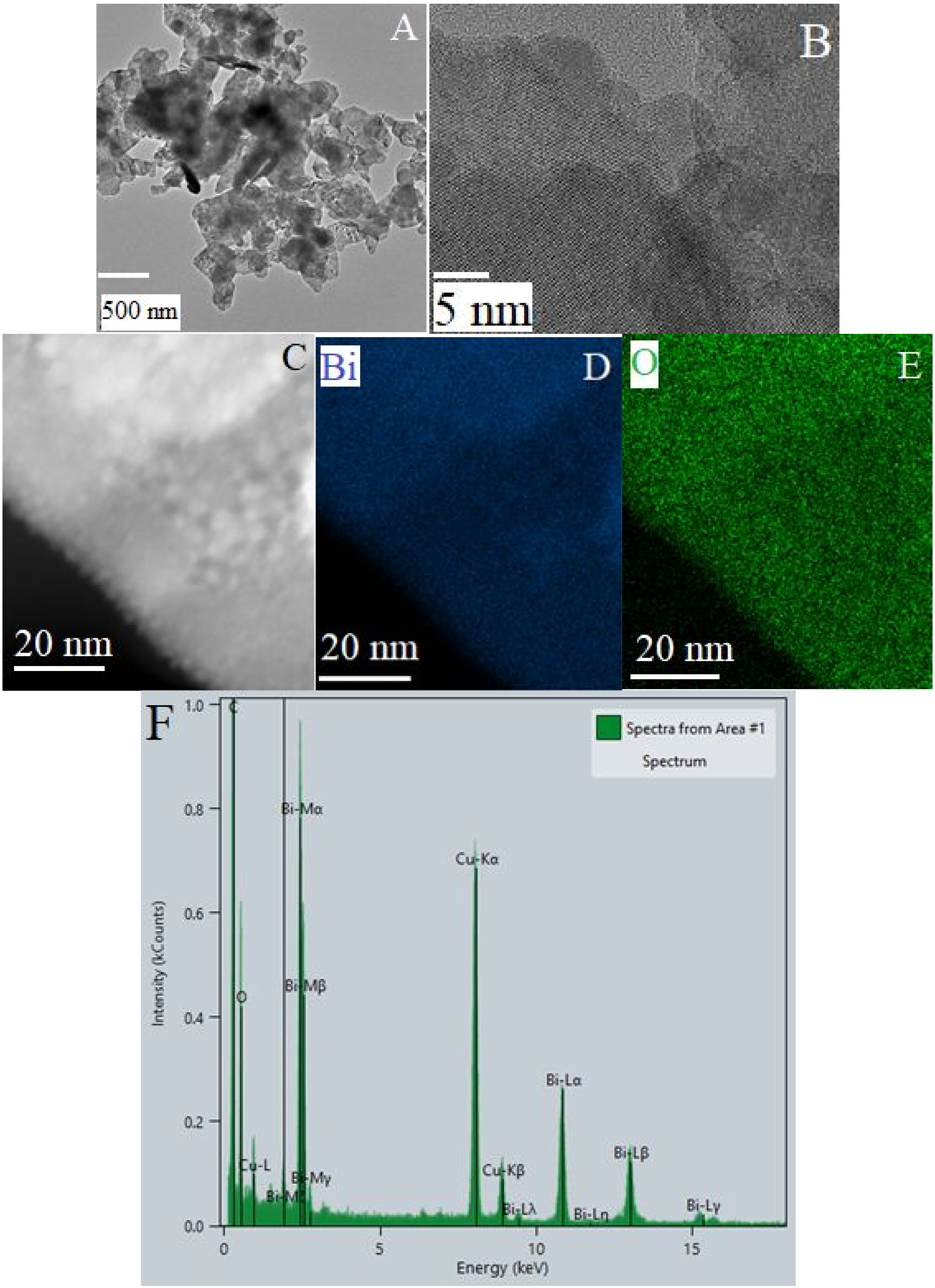

Figure S3. TEM images (A, B) and TEM-EDX elemental maps (C–E) of $Bi_2O_2CO_3$ as well as EDX spectrum (F) of $CuBi_2O_2CO_3$.

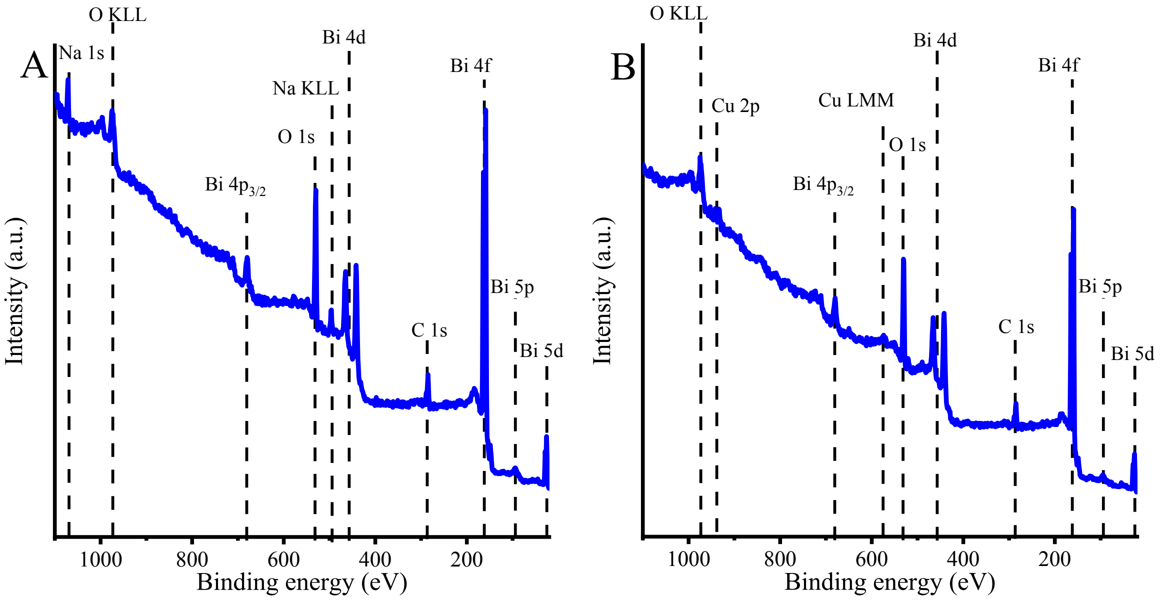

Figure S4. XPS survey scans of $Bi_2O_2CO_3$ (A) and Cu $Bi_2O_2CO_3$ (B).

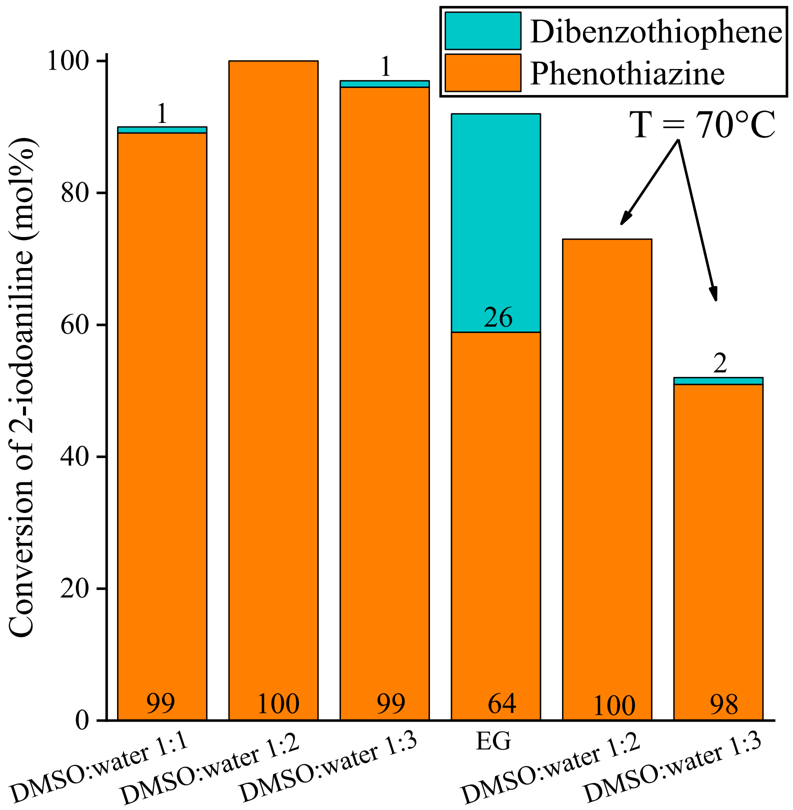

Figure S5. The effect of reaction temperature in the concerted C–S and C–N heterocyclization producing phenothiazine (see Scheme 1). Reaction conditions: 1 equiv. (0.25 M) 2-iodoaniline, 1.1 equiv. 2-bromothiophenol, 5 equiv. K_2CO_3, 5 mol% $CuBi_2O_2CO_3$, 70°C or 90°C, 72h.

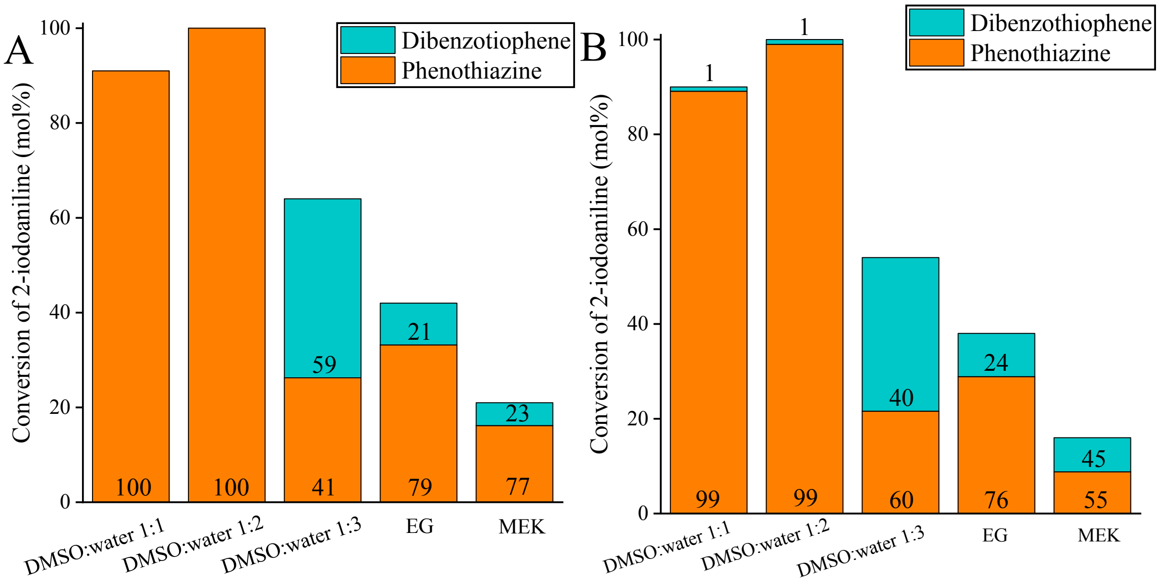

Figure S6. The effect of the quality of added base and the catalyst load in the concerted C–S and C–N heterocyclization producing phenothiazine (see Scheme 1). Reaction conditions: 1 equiv. (0.25 M) 2-iodoaniline, 1.1 equiv. 2-bromotiophenol, 2.5 equiv. K_2CO_3, 5 (A) or 2.5 (B) mol% $CuBi_2O_2CO_3$, 70°C (for EG and MEK) or 90°C, 72h.

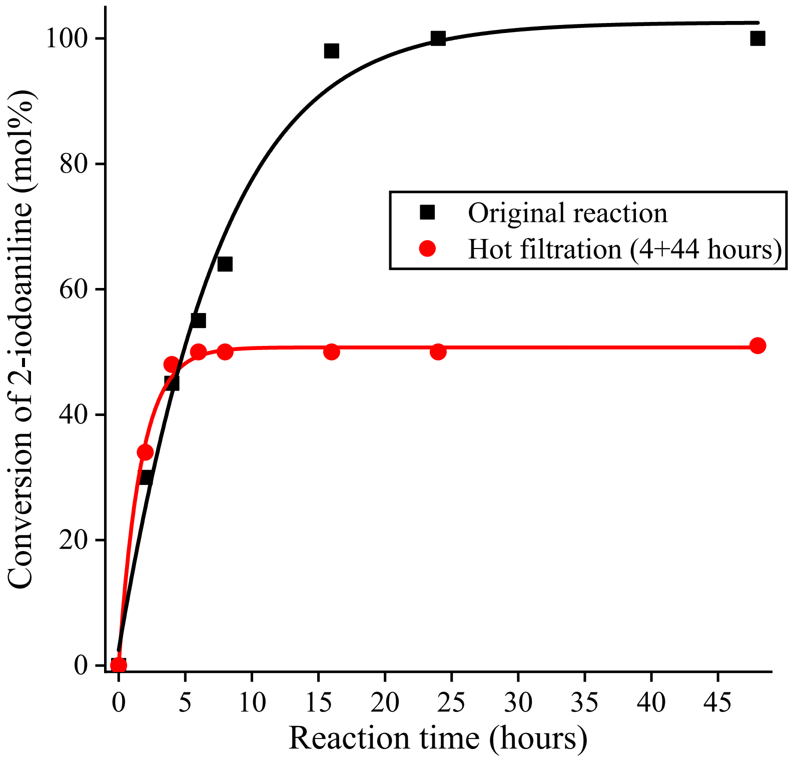

Figure S7. Hot filtration test in the concerted C–S and C–N heterocyclization producing phenothiazine (see Scheme 1): Conversions of 2-idoaniline. Reaction conditions: 1 equiv. (0.25 M) 2-iodoaniline, 1.1 equiv. 2-bromobenzenethiol, solvent: DMSO:water 1:2, 2.5 equiv. K_2CO_3, 2.5 mol% $CuBi_2O_2CO_3$, 90°C, 48h. (Hot filtration of used catalyst occurred after 4 hours.)

Table S3. Measured total pore volumes and specific surface areas as well as average pore diameters for the catalysts used.

Catalysts	Total pore volume (cm^3/g)	Surface area (m^2/g)	Average pore radius (nm)
$Bi_2O_2CO_3$	0.036	11.0	1.8
CuO	0.048	18.2	0.8
$CuO@Bi_2O_2CO_3$	0.056	21.8	1.1
$CuBi_2O_2CO_3$	0.039	12.1	1.7

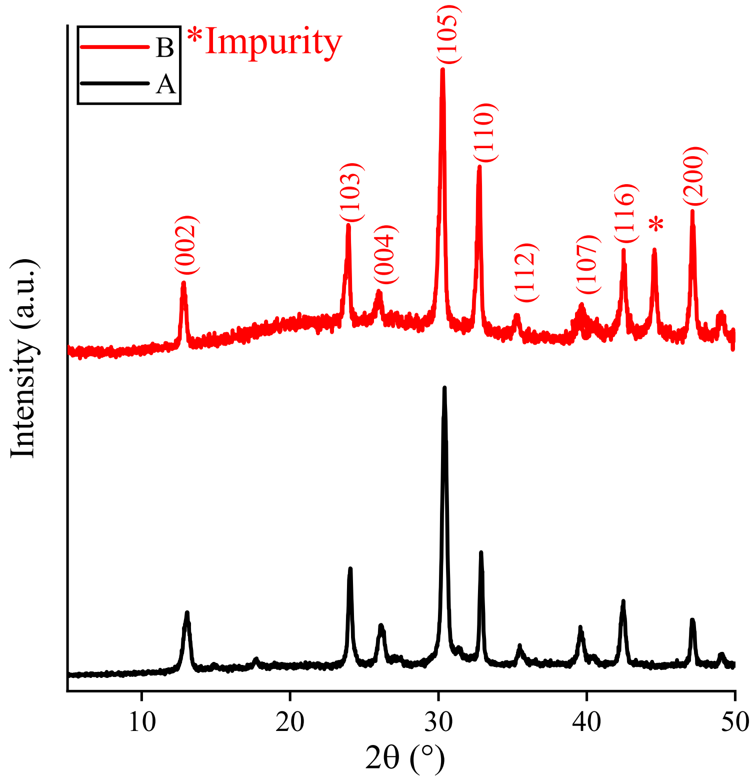

Figure S8. XRD patterns of (A) as-prepared and (B) used (after fifth recycling) $CuBi_2O_2CO_3$.

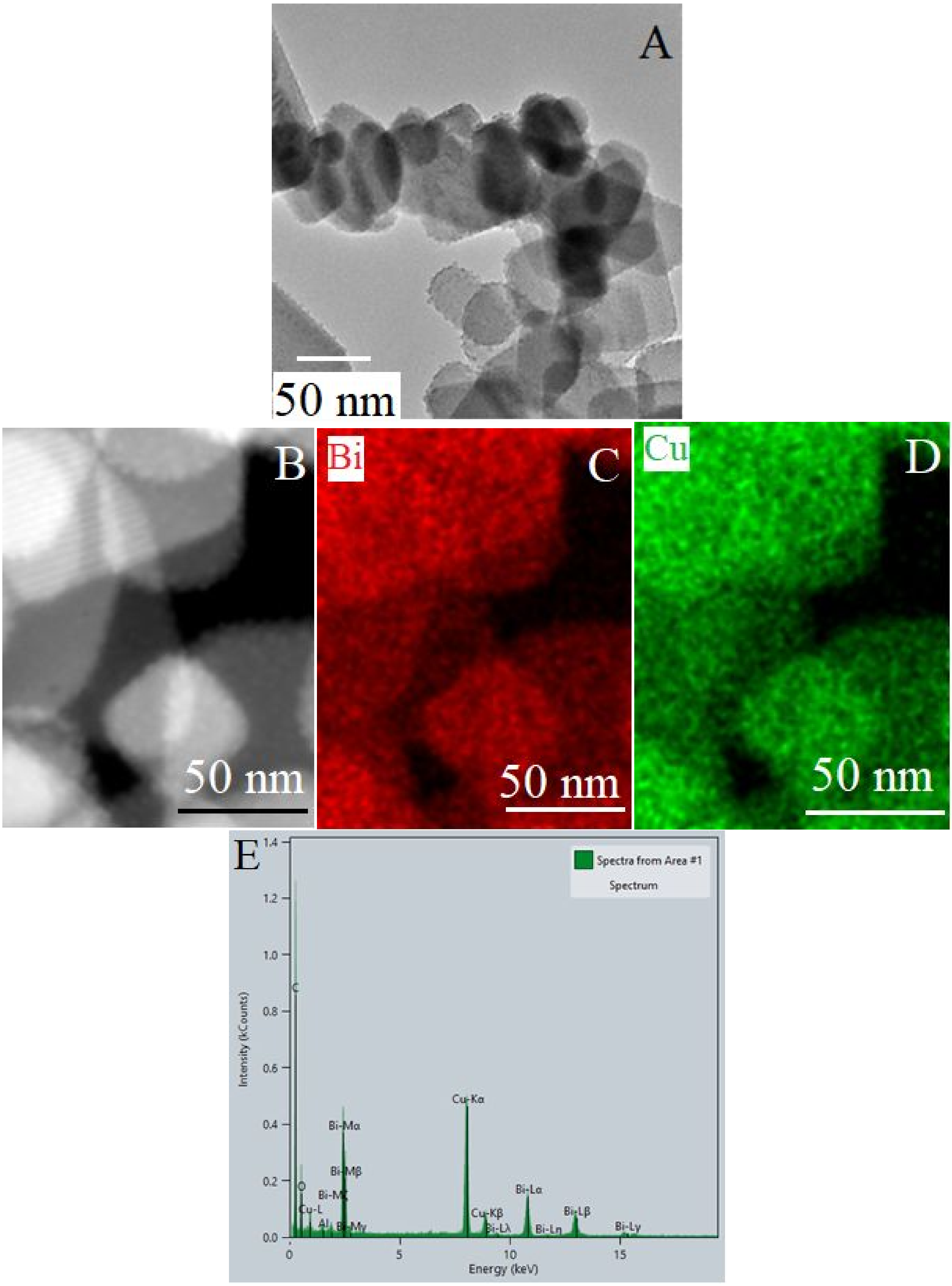

Figure S9. TEM image (A), TEM-EDX elemental maps (B–D) and EDX spectrum (E) of used $CuBi_2O_2CO_3$.

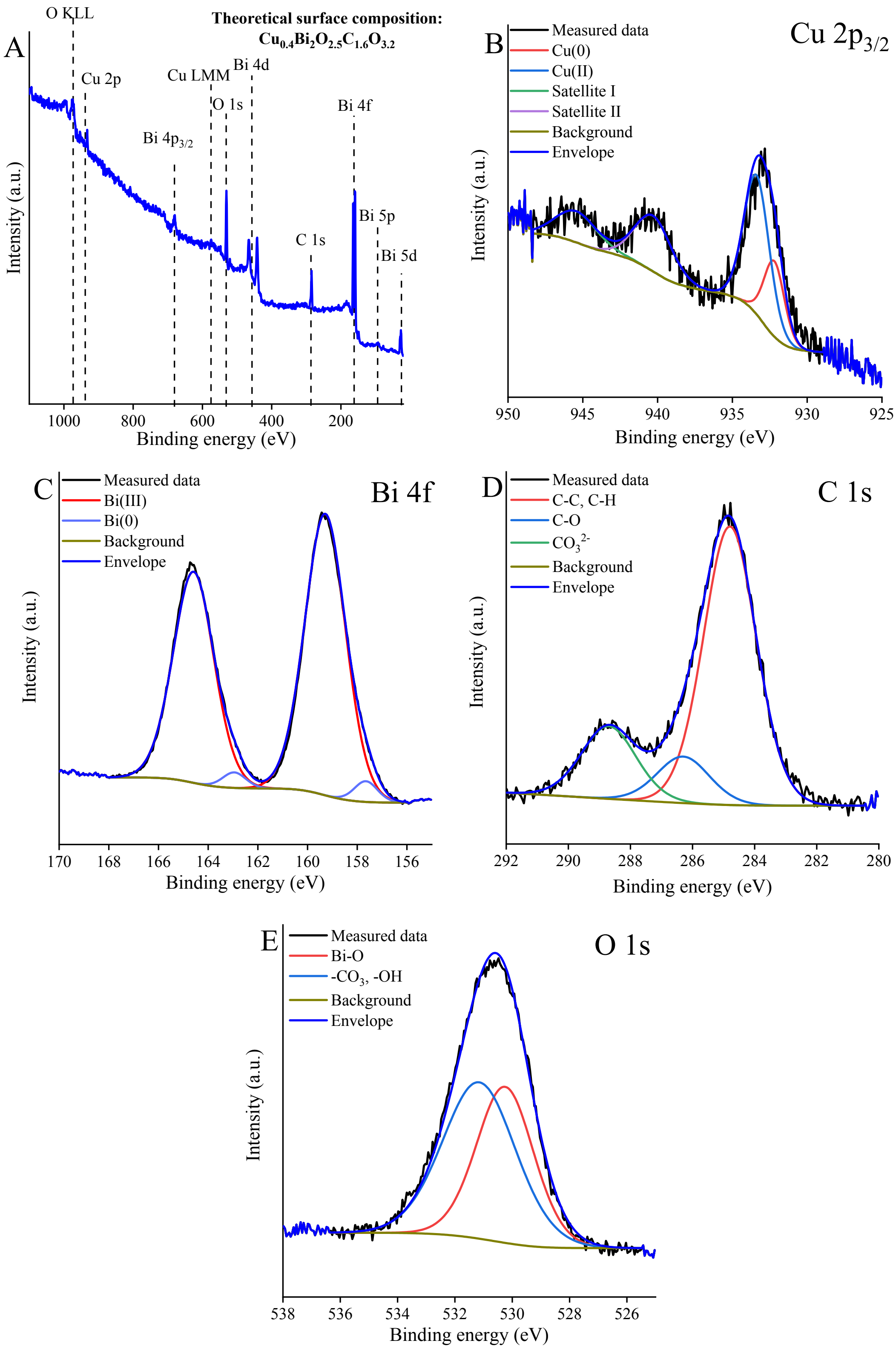

Figure S10. XPS survey scans (A) and Cu $2p_{3/2}$ (B), Bi 4f (C), C 1s (D) as well as O 1s (E) XP spectra of the used Cu $Bi_2O_2CO_3$ after the fifth reaction cycle.

S4. Identification of the produced phenothiazine and its derivatives by NMR spectroscopy

10H-phenothiazine:

^{1}H NMR (500 MHz, DMSO-d6) δ 7.20-7.16 (m, 4H), 7.13-7.09 (m, 2H), 7.00-6.97 (m, 2H).

^{13}C NMR (500 MHz, DMSO-d6) δ 127.33, 126.77, 121.48, 114.44, 114.24.

10H-phenothiazine-2-carbonitrile:

^{1}H NMR (500 MHz, DMSO-d6) δ 7.57-7.53 (m, 2H), 7.42-7.41 (d, 1H), 7.20-7.16 (m, 2H), 7.13-7.09 (m, 1H), 7.01—6.99 (d, 1H).

^{13}C NMR (125 MHz, DMSO-d6) δ 127.43, 127.33, 127.32, 125.34, 121.48, 117.30, 114.56.

2,4-dichloro-10H-phenothiazine:

^{1}H NMR (500 MHz, DMSO-d6) δ 7.64-7.63 (d, 2H), 7.33-7.31 (d, 2H), 7.22-7.20 (d, 2H), 7.19-7.16 (m, 2H) 7.13-7.09 (m, 2H), 7.01-6.99 (d, 2H).

^{13}C NMR (125 MHz, DMSO-d6) δ 127.42, 127.19, 123.21, 122.31, 115.65, 114.38.

2,4-dimethyl-10H-phenothiazine:

^{1}H NMR (500 MHz, DMSO-d6) δ 7-31-7.30 (d, 1H), 7.20-7.16 (t, 1H), 7.13-7.09 (t, 1H), 7.01-6.99 (m, 1H) 6.92-6.91 (m, 1H), 2.30 (s, 3H).

^{13}C NMR (125 MHz, DMSO-d6) δ 138.10, 125.52, 127.13, 123.98, 121.87, 115.62, 113.27, 21.06, 20.70.

2-chloro-10H-phenothiazine:

^{1}H NMR (500 MHz, DMSO-d6) δ 7.37-7.36 (d, 1H), 7.33-7.31 (d, 1H), 7.26 (d, 1H), 7.20-7.16 (m, 2H), 7.13-7.09 (m, 1H), 7.01-6.99 (d, 1H).

^{13}C NMR (125 MHz, DMSO-d6) δ 127.43, 127.33, 127.11, 121.48, 113.98, 113.88.

2-nitro-10H-phenothiazine:

^{1}H NMR (500 MHz, DMSO-d6) δ 8.14-8.10 (m, 2H), 7.69-7.68 (d, 1H), 7.20-7.16 (m, 2H), 7.13-7.09 (m, 1H), 7.01-7.00 (d, 1H).

^{13}C NMR (125 MHz, DMSO-d6) δ 128.29, 127.57, 127.33, 115.81, 114.62, 108.69.

2-chloro-4-fluoro-10H-phenothiazine:

^{1}H NMR (500 MHz, DMSO-d6) δ 7.41-7.40 (d, 1H), 7.23-7.18 (m, 1H), 7.17-7.13 (m, 1H), 7.12-7.10 (m, 1H), 7.01-7.00 (d, 1H).

^{13}C NMR (125 MHz, DMSO-d6) δ 134.09, 127.24, 127.13, 122.19, 115.80, 113.00, 112.96, 110.43, 110.25.
